# Primary-site variation in recorded metastatic patterns and survival associations in extrapulmonary poorly differentiated neuroendocrine carcinoma

**DOI:** 10.3389/fendo.2026.1975853

**Published:** 2026-10-22

**Authors:** Huadong Liang, Xiaoxin Wu, Xinjing Li, Qianqian Wu, Rongjin Qiu, Fengxiang Zheng

**Affiliations:** 1 Department of Gastroenterology, Longyan First Affiliated Hospital of Fujian Medical University, Longyan, Fujian, China; 2 Department of Pathology, Longyan First Affiliated Hospital of Fujian Medical University, Longyan, Fujian, China

**Keywords:** extrapulmonary, metastatic patterns, neuroendocrine carcinoma, prognosis, SEER

## Abstract

**Background:**

Extrapulmonary poorly differentiated neuroendocrine carcinoma (EP-PD-NEC) is aggressive and heterogeneous. Our primary objective was to assess primary-site differences in bone, brain, liver, and lung metastases recorded at diagnosis. Secondary analyses examined prognostic effect modification and the incremental predictive information in these variables.

**Methods:**

We analyzed Surveillance, Epidemiology, and End Results data from 2010–2023. Strict EP-PD-NEC was defined by ICD-O-3 codes 8013 and 8041–8045. Bone, brain, liver, and lung metastases recorded at diagnosis were evaluated using modified Poisson regression with individual-level heteroscedasticity-consistent standard errors. Overall survival (OS) was analyzed with multivariable Cox models containing primary-site-by-organ interactions. Associations identified in 2010–2017 were reassessed in 2018–2022. Four prespecified ridge-penalized Cox models were developed and temporally evaluated in distant-stage cases with complete four-organ status. Primary prevalence analyses included patients with known status for the organ analyzed; sensitivity analyses recoded unknown status as negative or used multiple imputation.

**Results:**

Among 6,044 eligible patients, 5,820 (96.3%) had complete four-organ information and 2,356 had at least one recorded organ involved. Recorded metastatic prevalence differed across six primary-site groups for every organ (all false-discovery-rate-adjusted P<0.001). Versus genitourinary primaries, pancreatic primaries had higher adjusted prevalence of liver (prevalence ratio [PR] 3.80, 95% confidence interval [CI] 3.39–4.25) and brain metastases (PR 2.86, 95% CI 1.75–4.66). An increasing recorded four-organ count was associated with shorter OS. The global primary-site-by-organ interaction was significant (likelihood-ratio χ²=43.49, 20 df, P = 0.00176), but the full interaction matrix added negligible temporal predictive value (ΔC-index=0.00169). In 1,374 later-period patients, the spectrum model had a C-index of 0.607 (95% CI 0.588–0.624), a 12-month area under the curve of 0.672 (95% CI 0.639–0.706), and a Brier score of 0.196 (95% CI 0.185–0.207). Compared with the four-organ-count model, paired bootstrap differences were 0.0085 (95% CI 0.0035–0.0139) for the C-index, 0.0119 (95% CI 0.0033–0.0231) for 12-month AUC, and −0.0020 (95% CI −0.0038 to −0.0004) for the Brier score. Missing-status analyses produced similar prevalence estimates.

**Conclusions:**

Recorded metastatic patterns differed by primary site in EP-PD-NEC. Organ identity added little predictive information beyond the recorded four-organ count, and site-by-organ interactions yielded negligible improvement. Overall discrimination remained modest.

## 1 Introduction

Neuroendocrine neoplasms encompass biologically distinct tumors that share neuroendocrine differentiation but differ substantially in morphology, molecular alterations, clinical behavior, and treatment responsiveness. Contemporary classification separates well-differentiated neuroendocrine tumors from poorly differentiated neuroendocrine carcinomas (NECs), the latter comprising small-cell and large-cell phenotypes and generally displaying rapid progression and poor survival (1). This distinction is clinically consequential: high proliferative activity alone does not make a well-differentiated grade 3 tumor equivalent to a poorly differentiated NEC (2, 3).

Extrapulmonary poorly differentiated NEC (EP-PD-NEC) is rare and can arise in nearly any organ. Its rarity has limited prospective evidence, and management has historically been extrapolated from small-cell lung cancer. Platinum–etoposide remains the usual first-line regimen for metastatic disease, although evidence is largely retrospective and responses are often short lived (3–7). Population-based studies have identified differences in stage distribution and survival by extrapulmonary primary site and morphology (8). These differences raise questions about how metastatic patterns and their survival associations vary across primary sites.

Metastasis is shaped by tumor, anatomical, diagnostic, and organ-level factors (9, 10). Recorded metastatic patterns reflect both disease characteristics and clinical ascertainment. The extent of primary-site variation in bone, brain, liver, and lung involvement in EP-PD-NEC remains incompletely characterized. Whether the survival associations of these metastases also differ by primary site is uncertain.

Whether metastatic organ identity provides prognostic information beyond the number of involved organs also remains uncertain. Differences in metastatic distribution may not translate into better survival prediction.

We examined primary-site differences in metastatic patterns at diagnosis in EP-PD-NEC. Secondary analyses assessed site-specific survival associations and the incremental predictive value of metastatic information.

## 2 Materials and methods

### 2.1 Data source and study design

We performed a retrospective population-based cohort study using the SEER*Stat Database: Incidence - SEER Research Data, 17 Registries, Nov 2025 Sub (2000-2023), linked to County Attributes - Time Dependent (1990-2024) Income/Rurality, 1969–2024 Counties (11). Case-listing data were extracted with SEER*Stat software version 9.0.43.0 (12). We included diagnoses from 2010 because all four site-specific metastasis variables required for the study were available from that year. The export contained tumors diagnosed from January 1, 2010, through December 31, 2023. SEER records tumors rather than unique persons; accordingly, the main analysis was restricted to the first malignant primary to reduce within-person duplication and ambiguity in assigning outcomes.

We evaluated organ-specific prevalence in the eligible cohort and prognostic effect modification in the core metastatic-spectrum cohort. Prognostic models were developed separately among distant-stage cases with complete four-organ status. Reporting followed STROBE for the observational analyses and TRIPOD+AI principles for model development and internal temporal assessment (13).

### 2.2 Cohort definition

Strict EP-PD-NEC was identified using ICD-O-3 morphology codes 8013 and 8041–8045, encompassing large-cell and small-cell NEC phenotypes. Code 8246 (neuroendocrine carcinoma, not otherwise specified) was excluded from the primary definition because it included substantial grade heterogeneity in the source data; it was admitted only in a sensitivity analysis when accompanied by high-grade evidence. Pulmonary primary sites were excluded.

Eligible tumors were malignant, microscopically confirmed, not identified solely by death certificate or autopsy, compatible with invasive disease, the patient’s first malignant primary, and accompanied by valid survival time and vital status. Unknown primary sites were excluded because the central exposure was primary-site group. Patients younger than 18 years were excluded. The complete cohort-construction sequence is shown in **Supplementary Figure 1**.

Primary sites were grouped *a priori* into six categories: genitourinary, gynecologic, colorectal, pancreatic, upper gastrointestinal, and other extrapulmonary sites. Less common primary sites were pooled in the other category to reduce sparsity in site-by-organ analyses; their component distributions were also tabulated separately. Detailed ICD-O-3 topography mappings are provided in **Supplementary Table S1** and the analysis code.

### 2.3 Metastatic organs and analysis populations

SEER site-specific metastasis variables identified bone, brain, liver, and lung involvement at diagnosis. For organ-specific prevalence analyses, the denominator for each organ consisted of patients recorded as either positive or negative for that organ; unknown status was excluded rather than recoded as absence. Analyses of metastatic combinations required complete status for all four organs.

The core metastatic-spectrum cohort comprised patients with complete status for bone, brain, liver, and lung and at least one of these organs recorded as positive. This cohort was used for analyses of the recorded four-organ count, metastatic-pattern survival, and primary-site-by-organ survival interactions. Patients with distant disease confined to peritoneal, distant nodal, or other unrecorded sites were not included in this cohort.

The prognostic-model cohort comprised all strict EP-PD-NEC cases with SEER distant stage at diagnosis, complete four-organ status, first malignant primary, and valid OS. Unlike the core metastatic-spectrum cohort, it retained patients whose four recorded organs were all negative. Cases diagnosed in 2023 were excluded from model development and temporal assessment because follow-up was immature. Analysis populations are summarized in **Supplementary Methods** (Analysis populations).

### 2.4 Outcomes and covariates

For survival analyses, the outcome was OS, defined from diagnosis to death from any cause or last follow-up. SEER cause-specific death classification was used in a sensitivity analysis. Baseline covariates were age at diagnosis, sex, race/ethnicity recode, year of diagnosis, morphology (small-cell vs large-cell), primary-site group, and organ-specific metastasis status. Race/ethnicity was included as a registry covariate for adjustment.

Predictors were restricted to information available at diagnosis; treatment variables were excluded. Surgery, radiotherapy, and chemotherapy were analyzed separately as descriptive measures. Treatment frequencies were summarized by calendar period. Stage was not included as a predictor because model eligibility was restricted to distant-stage disease, leaving no stage variation to estimate.

### 2.5 Descriptive and recorded metastatic-pattern analyses

Continuous variables were summarized with medians and interquartile ranges (IQRs), and categorical variables with counts and percentages. Primary-site differences in the prevalence of each metastatic organ were initially assessed using Pearson χ² tests. P values for the four global organ tests were controlled using the Benjamini–Hochberg false discovery rate (FDR) procedure (14).

Adjusted prevalence ratios (PRs) were estimated separately for each organ using modified Poisson regression with individual-level heteroscedasticity-consistent robust standard errors (15). The independent variable of interest was primary-site group, with genitourinary primaries as the reference. Models adjusted for age, sex, race/ethnicity, diagnosis year, and morphology. Other metastatic organs were not included because the estimand was the marginal site-specific prevalence of the target organ rather than a conditional association given co-metastases. Registry identifiers were unavailable in the standard SEER Research case-listing export; registry-level clustering was not modeled.

Unknown status was excluded from the primary organ-specific analyses. We quantified organ-specific and patient-level missingness, compared patients with complete and incomplete four-organ status, and performed two sensitivity analyses: (i) recoding unknown organ status as negative, reflecting a common registry convention, and (ii) 20 chained-equation multiple imputations using demographics, diagnosis year, morphology, primary site, stage, and the other organ indicators. Estimates were combined using Rubin’s rules. A separate diagnostic run compared 10 and 50 updates in 20 chains and assessed mixing of imputed organ frequencies and Monte Carlo error of pooled log-PRs.

The recorded four-organ count was categorized as one, two, three, or four positive organs in the core metastatic-spectrum cohort. The most frequent mutually exclusive combinations were tabulated. Kaplan–Meier methods estimated median OS and survival at fixed time points. Fixed-horizon survival was not reported beyond the last observed follow-up in a stratum when the final Kaplan–Meier estimate remained above zero.

### 2.6 Prognostic effect modification

The main-effects Cox model included age, sex, race/ethnicity, diagnosis year, morphology, six-category primary site, and indicators for bone, brain, liver, and lung metastases. The interaction model added all primary-site-by-organ product terms. A likelihood-ratio test comparing these nested models provided the global test of effect modification. Site-specific organ hazard ratios (HRs) were derived as linear combinations of the relevant main and interaction coefficients. FDR-adjusted P values for displayed site-specific effects were treated as exploratory. Sparse-data diagnostics tabulated positive and negative organ records and deaths within each primary-site group. Interaction coefficients were compared over an exploratory ridge-penalty grid without selecting a new inferential model.

The proportional-hazards assumption was evaluated using Schoenfeld residuals. An exploratory piecewise Cox analysis used a six-month split to examine time variation in site-specific liver associations; model specifications are given in **Supplementary Methods**. A 12-month mortality logistic sensitivity analysis included patients who died within 12 months or were followed for at least 12 months; earlier-censored survivors were excluded. Additional sensitivity analyses used cancer-specific death as the outcome and excluded patients with positive organ records but localized or regional SEER stage. Residual proportionality was also tested after the piecewise extension.

The interaction analysis was repeated after allowing all malignant primaries, excluding primary liver tumors, and adding morphology code 8246 when accompanied by high-grade evidence. An exploratory lower-dimensional model included five liver-by-primary-site interaction terms with all prespecified main effects and other organ indicators. This 5-df interaction was evaluated in the full core metastatic-spectrum cohort and separately in 2010–2017 and 2018–2022.

### 2.7 Temporal reassessment of metastatic patterns and prognostic effects

Eligible data were divided into an earlier discovery/development period (2010–2017) and a nonoverlapping later assessment period (2018–2022); 2023 was excluded because follow-up was immature. The split provided similarly sized prediction cohorts while retaining follow-up for fixed-horizon assessment. Associations meeting FDR<0.05 in the earlier period were re-estimated in the later period, with direction and 95% CIs reported separately. The global 20-df interaction and an exploratory focused 5-df liver interaction were assessed in both periods. Performance and treatment records were additionally summarized for 2018–2019 and 2020–2022.

### 2.8 Prespecified prognostic models

Four candidate ridge-penalized Cox models used the following predictor sets:

- M0 (Basic): age modeled using a four-knot restricted cubic spline, sex, and morphology;
- M1 (Site): M0 plus six-category primary site;
- M2 (Four-organ count): M1 plus the number of involved recorded organs among bone, brain, liver, and lung (0, 1, 2, or ≥3);
- M3 (Spectrum): M1 plus individual bone, brain, liver, and lung indicators.

The raw organ count was derived from the four binary organ indicators and grouped as 0, 1, 2, or ≥3 for M2. M2 and M3 compared grouped organ count with organ identity using separate models. Age knots were fixed from the development data at 40, 60, 70.4, and 85 years. No univariable screening, stepwise selection, treatment variables, or primary-site-by-organ interactions were used in these parsimonious models.

### 2.9 Model development and temporal assessment

Models were trained in patients diagnosed during 2010–2017 and evaluated without refitting in patients diagnosed during 2018–2022. Ridge penalties were selected in the development cohort by five-fold cross-validation minimizing the mean inverse-probability-of-censoring-weighted (IPCW) Brier score at 6, 12, and 24 months. Coefficient shrinkage was quantified against unpenalized fits using the same standardized design, alongside changes in the standard deviation of the linear predictor. Schoenfeld tests in identifiable unpenalized analogues were used to examine proportionality of the prediction-model predictor sets; these were not formal tests of the penalized estimators.

Discrimination was assessed with Harrell’s concordance index, Uno’s C at 24 months, and cumulative/dynamic area under the time-dependent receiver-operating-characteristic curve (AUC) at 6, 12, and 24 months (16, 17). Prediction error was assessed with IPCW Brier scores (18). These assessments covered discrimination, calibration, and prediction error (13, 19–21). Calibration was described by calibration-in-the-large, calibration slope, comparison of mean predicted with Kaplan–Meier-observed risk, and grouped calibration plots. Ridge penalty strength was selected in the development cohort and remained fixed during later-period evaluation. Patient-level bootstrap resampling of the later cohort (300 repetitions) estimated 95% CIs for performance metrics using predictions from the fixed fitted models; models were not refitted and penalty strength was not retuned within these resamples. Bootstrap optimism was estimated separately in the development cohort by refitting the selected model with the fixed penalty in each resample. To quantify incremental value beyond the recorded four-organ count, an additional 500 paired bootstrap resamples of the later cohort used identical patient indices for M2 and M3 and estimated confidence intervals for differences in Harrell C, 12-month AUC, and 12-month Brier score.

The protocol compared M3 with M1 using later-period performance to select the model for risk-group and subgroup analyses. Selection criteria and their implementation are described in **Supplementary Methods M2** and **M3** represented alternative encodings of metastatic information. Their paired comparison estimated the incremental predictive value of organ identity relative to the recorded four-organ count. Development-set tertiles of predicted 12-month mortality were applied unchanged to the later cohort. Subgroup performance was summarized descriptively.

### 2.10 Statistical software

Analyses were performed using Python version 3.12.14. Major packages included statsmodels, lifelines, scikit-learn, and scikit-survival (version 0.28.0); package versions are listed in the reproducibility archive. Additional piecewise Cox and stage-discordance sensitivity analyses used R version 4.6.1 and the survival package version 3.8.6. All hypothesis tests were two sided. Unless otherwise specified, P<0.05 was considered statistically significant.

## 3 Results

### 3.1 Cohort construction and baseline characteristics

The SEER export contained 121,264 tumor records. Application of the strict histology and eligibility criteria yielded 6,044 patients with EP-PD-NEC (**Supplementary Figure 1**). Of these, 4,826 (79.8%) had small-cell morphology and 1,218 (20.2%) had large-cell morphology. Median age was 67 years (IQR 57–75), and 57.0% were male. Primary sites were genitourinary in 35.3%, gynecologic in 15.2%, colorectal in 15.0%, pancreatic in 6.8%, upper gastrointestinal in 9.6%, and other extrapulmonary sites in 18.1%. The other-site group comprised head and neck (n=431; 39.3%), hepatobiliary (n=294; 26.8%), residual uncommon sites (n=154; 14.1%), breast (n=125; 11.4%), and small-bowel or appendiceal primaries (n=92; 8.4%). Component-specific recorded metastatic patterns are reported in **Supplementary Table S2**. Distant-stage disease was recorded in 52.0%, and first-course chemotherapy was recorded as administered in 67.8% (**Table 1**).

**Table 1:** Study populations and selected baseline characteristics.

Characteristic	Value
Source tumor records	121,264
Strict eligible EP-PD-NEC cohort	6,044
Small-cell morphology	4,826 (79.8%)
Large-cell morphology	1,218 (20.2%)
Age, median (IQR), years	67 (57–75)
Male sex	57.0%
Genitourinary primary	35.3%
Gynecologic primary	15.2%
Colorectal primary	15.0%
Pancreatic primary	6.8%
Upper gastrointestinal primary	9.6%
Other primary	18.1%
SEER distant stage	52.0%
Distant stage, genitourinary	930/2,131 (43.6%)
Distant stage, gynecologic	434/920 (47.2%)
Distant stage, colorectal	571/908 (62.9%)
Distant stage, pancreatic	339/410 (82.7%)
Distant stage, upper gastrointestinal	367/579 (63.4%)
Distant stage, other	500/1,096 (45.6%)
Chemotherapy recorded as yes	67.8%
Complete four-organ status	5,820
Core metastatic-spectrum cohort	2,356 (2,087 deaths)
Complete distant-stage model cohort	3,018 (2,615 deaths)
Model development cohort, 2010–2017	1,297 (1,241 deaths)
Model later-period temporal-assessment cohort, 2018–2022	1,374 (1,214 deaths)

Values are counts, percentages, n (%), or median (IQR), as labeled. Baseline percentages refer to the strict eligible cohort (n=6,044), except site-specific distant-stage percentages, which use the corresponding primary-site denominator. Death counts are shown separately for survival-analysis cohorts. EP-PD-NEC, extrapulmonary poorly differentiated neuroendocrine carcinoma; IQR, interquartile range; SEER, Surveillance, Epidemiology, and End Results.

Complete bone, brain, liver, and lung status was available for 5,820 patients (96.3%); 224 patients (3.7%) had at least one unknown organ status. Organ-specific missingness ranged from 2.1% for liver to 2.9% for lung (**Supplementary Table S3**). Patients with incomplete status were older (mean 67.5 vs 65.3 years), were diagnosed earlier (mean year 2015.6 vs 2017.5), and more often died during follow-up (91.5% vs 73.8%); the complete comparison, including site and stage distributions, is provided in **Supplementary Table S4**. The core metastatic-spectrum cohort contained 2,356 patients with at least one of these four organs positive, of whom 2,087 died. The prognostic-model cohort contained 3,018 distant-stage patients, including 670 with all four recorded organs negative. After excluding 2023 diagnoses, 1,297 patients (1,241 deaths) formed the 2010–2017 development cohort and 1,374 (1,214 deaths) formed the 2018–2022 later-period cohort. The core cohort included eight organ-positive cases recorded as localized (n=1) or regional (n=7) stage; these were absent from the distant-stage prediction cohort. The 347 prediction-cohort cases diagnosed in 2023 were excluded from temporal modeling.

### 3.2 Primary-site variation in recorded metastatic patterns

The prevalence of every recorded metastatic organ differed across the six primary-site groups (**Figure 1A**; **Table 2**). Global P values were approximately 1.00×10^-^²^4^ for bone, 2.81×10^-5^ for brain, 3.20×10^-^¹^66^ for liver, and 1.59×10^-4^ for lung; all remained significant after FDR correction.

**Figure 1:**
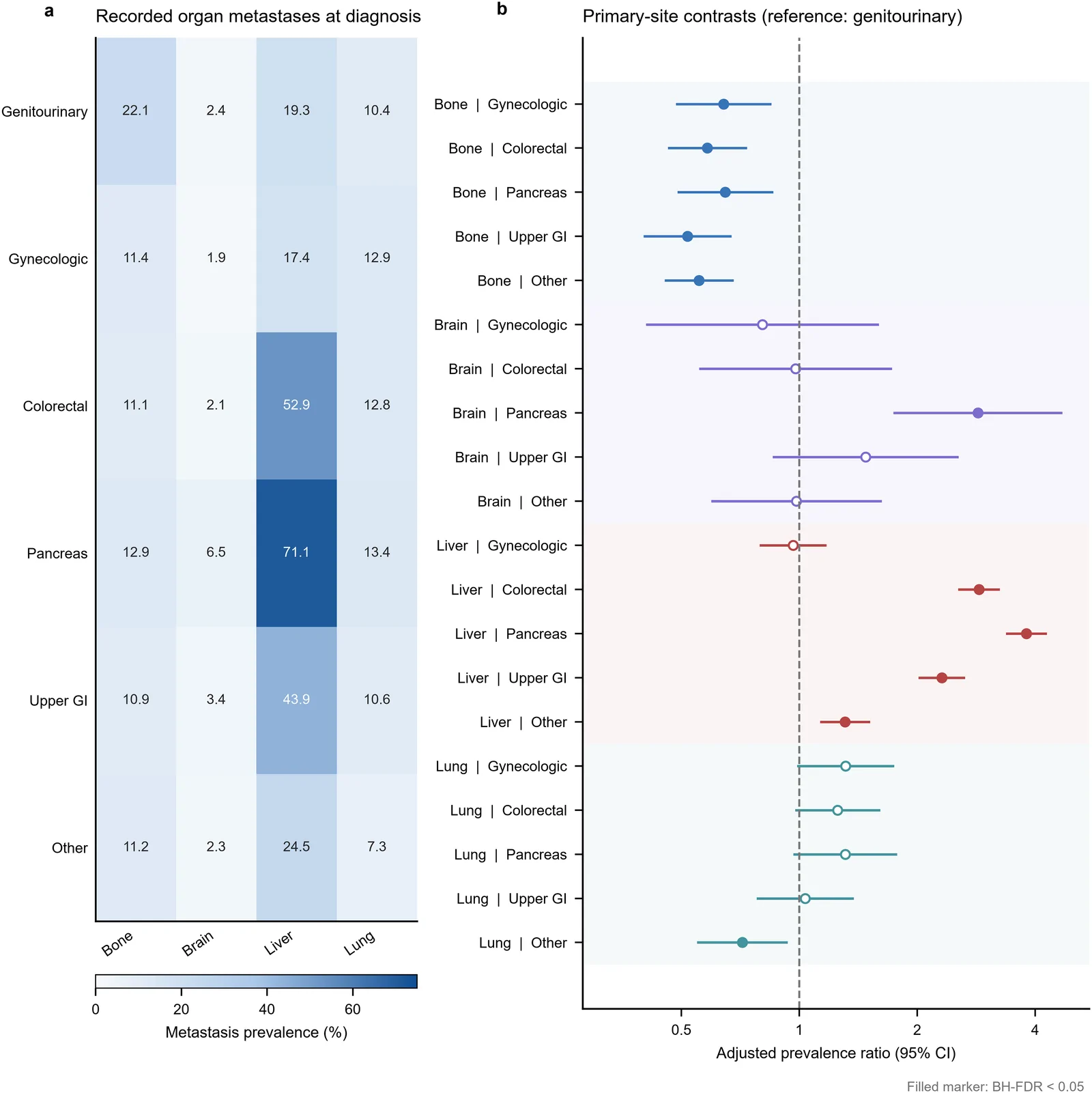
Primary-site variation in recorded metastatic prevalence in extrapulmonary poorly differentiated neuroendocrine carcinoma. **(A)** Prevalence of bone, brain, liver, and lung metastases recorded at diagnosis among patients with known status for the corresponding organ. Cell values are percentages; organ-specific denominators vary because unknown values are excluded rather than classified as negative. Across the six primary-site groups, global Pearson χ² tests are significant for bone (P = 1.00×10^-^²^4^), brain (P = 2.81×10^-5^), liver (P = 3.20×10^-^¹^66^), and lung (P = 1.59×10^-4^) metastases; all remain significant after Benjamini–Hochberg correction. **(B)** Adjusted prevalence ratios and 95% CIs from modified Poisson models with individual-level robust standard errors, adjusted for age, sex, race/ethnicity, diagnosis year, and morphology. Genitourinary primary is the reference. Filled markers indicate BH-FDR<0.05 across displayed contrasts. CI, confidence interval; BH-FDR, Benjamini–Hochberg false discovery rate; GI, gastrointestinal.

**Table 2:** Prevalence of recorded organ metastases by primary-site group.

Primary-site group	Bone	Brain	Liver	Lung
Genitourinary	462/2,089 (22.1%)	51/2,082 (2.4%)	404/2,091 (19.3%)	216/2,076 (10.4%)
Gynecologic	103/903 (11.4%)	17/901 (1.9%)	157/902 (17.4%)	116/898 (12.9%)
Colorectal	98/884 (11.1%)	19/887 (2.1%)	472/892 (52.9%)	113/883 (12.8%)
Pancreatic	51/394 (12.9%)	26/397 (6.5%)	285/401 (71.1%)	53/396 (13.4%)
Upper gastrointestinal	61/559 (10.9%)	19/556 (3.4%)	247/563 (43.9%)	59/557 (10.6%)
Other	119/1,065 (11.2%)	24/1,066 (2.3%)	262/1,071 (24.5%)	77/1,060 (7.3%)

Cells show positive/known-status denominator (percentage); unknown values were excluded rather than classified as negative.

The greatest contrast involved liver metastasis. Liver involvement was recorded in 71.1% of pancreatic, 52.9% of colorectal, and 43.9% of upper gastrointestinal primaries, compared with 19.3% of genitourinary primaries. After adjustment, the corresponding PRs versus genitourinary primaries were 3.80 (95% CI 3.39–4.25) for pancreatic, 2.88 (95% CI 2.56–3.23) for colorectal, and 2.31 (95% CI 2.03–2.63) for upper gastrointestinal tumors (**Figure 1B**).

Brain metastasis was uncommon overall, but pancreatic primaries had the highest recorded prevalence (6.5%) and an adjusted PR of 2.86 (95% CI 1.75–4.66) relative to genitourinary primaries. Conversely, bone involvement was most frequent in genitourinary primaries (22.1%); adjusted bone-metastasis prevalence in every other site group was approximately 35%–48% lower (PR range, approximately 0.52–0.65). Lung differences were smaller, ranging from 7.3% in other sites to 13.4% in pancreatic primaries.

Missing-status sensitivity analyses did not materially change the principal contrasts. For pancreatic versus genitourinary liver metastasis, the PR was 3.80 (95% CI 3.39–4.25) among patients with known liver status, 3.77 (95% CI 3.36–4.22) when unknown values were treated as negative, and 3.79 (95% CI 3.38–4.24) after multiple imputation. Corresponding colorectal liver PRs were 2.87, 2.86, and 2.85; upper gastrointestinal liver PRs were 2.31, 2.28, and 2.28 (**Supplementary Table S5**). In a separate 20-chain diagnostic run, extending imputation from 10 to 50 updates changed the 20 site-specific PRs by at most 2.27% in relative terms; the pancreatic liver PR changed from 3.783 to 3.788 (**Supplementary Tables S5B, C**).

### 3.3 Recorded four-organ count and metastatic patterns

Among the 2,356 patients in the core metastatic-spectrum cohort, 1,543 had one recorded organ, 611 had two, 185 had three, and 17 had all four organs involved. Median OS progressively shortened from 6.70 months for one organ to 5.32, 4.63, and 4.40 months for two, three, and four organs, respectively (**Figure 2A**). Twelve-month OS estimates were 29.7%, 20.9%, and 8.8% for one, two, and three organs, respectively. In the four-organ group, the longest observed follow-up was 9.72 months; no patient remained under observation at 12 months, so a 12-month OS estimate was not reported.

**Figure 2:**
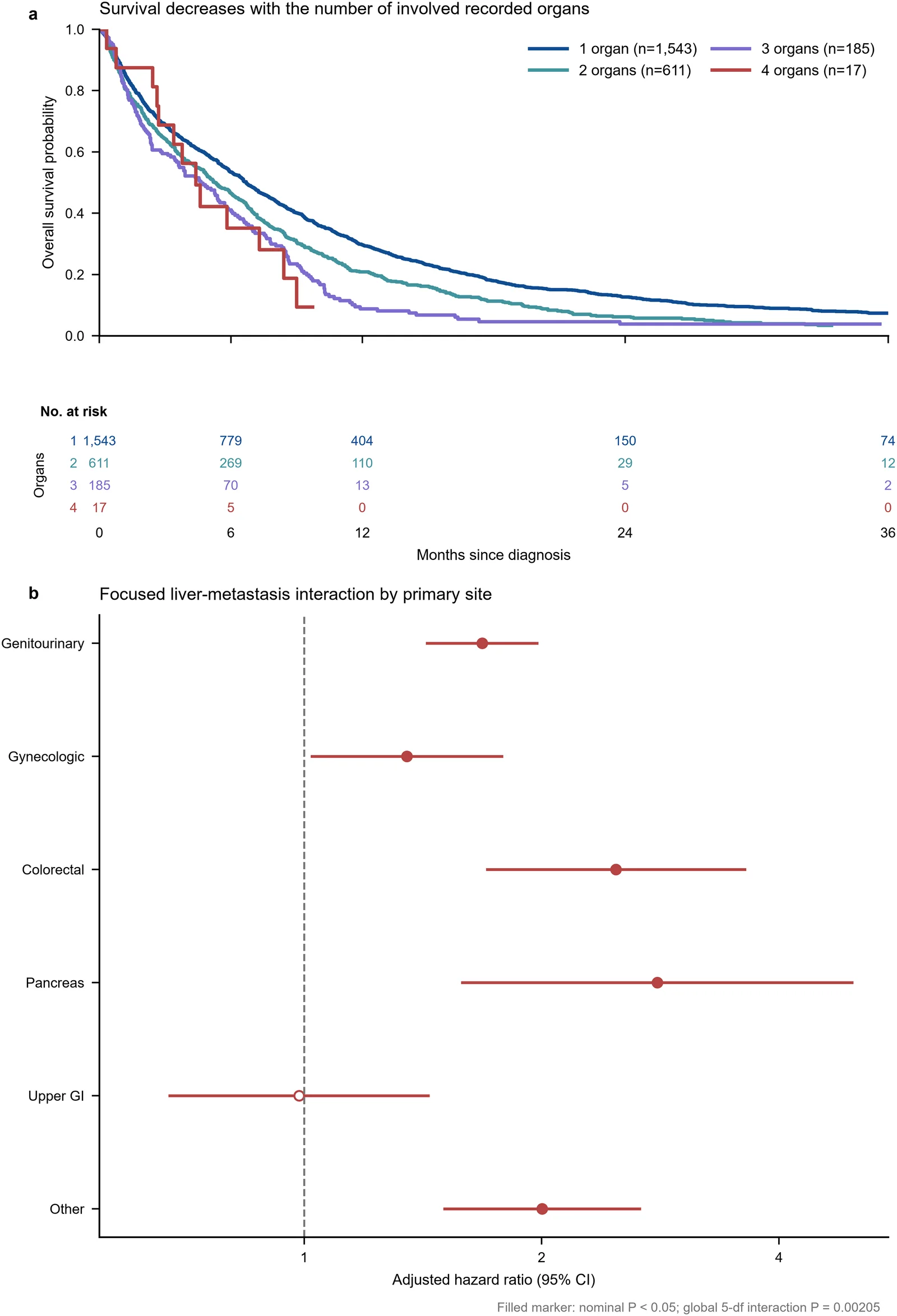
Recorded four-organ count and the focused liver-metastasis interaction. **(A)** Kaplan–Meier OS among 2,356 patients with complete bone, brain, liver, and lung status and at least one positive recorded organ, stratified by number of involved organs. Group sizes are n=1,543, 611, 185, and 17 for one, two, three, and four organs. The four-organ group had no patients under observation at 12 months; its curve ends at the last observed follow-up (9.72 months). **(B)** Adjusted liver-metastasis HRs and 95% CIs within each primary-site group from the focused Cox interaction model. Filled markers indicate nominal P<0.05 for the stratum-specific liver association. The focused liver-by-primary-site likelihood-ratio test gives χ²=18.84 with 5 df (P = 0.00205). The complete 24-estimate interaction matrix is shown in **Supplementary Figure 2**. Models adjusted for age, sex, race/ethnicity, diagnosis year, morphology, primary site, all four organ indicators, and liver-by-site interactions. OS, overall survival; HR, hazard ratio; CI, confidence interval; df, degrees of freedom; GI, gastrointestinal.

Liver involvement alone among the four recorded organs was the most common pattern (n=1,038; median OS 5.82 months; 12-month OS 25.0%), followed by bone plus liver (n=308; 5.72 months; 20.6%) and bone only (n=302; 9.99 months; 40.2%). Lung involvement alone among these organs had a median OS of 8.18 months and 12-month OS of 37.0%. Median OS was 3.45 months for liver plus lung disease and 4.53 months for bone plus liver plus lung disease.

### 3.4 Primary-site-specific survival associations

Adding all primary-site-by-organ terms improved model fit over the main-effects Cox model (likelihood-ratio χ²=43.49, 20 df, P = 0.00176; **Supplementary Figure 2**).

Selected adjusted survival associations included liver metastasis in genitourinary primaries (HR 1.65, 95% CI 1.40–1.95), bone metastasis in colorectal primaries (HR 1.36, 95% CI 1.07–1.75), and liver metastasis in colorectal (HR 2.35, 95% CI 1.59–3.47) and pancreatic primaries (HR 3.02, 95% CI 1.64–5.58). Within the other-site group, bone, liver, and lung involvement had HRs of 1.63 (95% CI 1.27–2.09), 2.18 (95% CI 1.58–2.99), and 1.52 (95% CI 1.11–2.07), respectively. The adjusted liver HR was 1.03 among upper gastrointestinal primaries.

The full interaction model contained 37 coefficients. Its early-period cohort included 986 patients and 957 deaths; brain-positive strata contained 5–22 patients and 4–22 deaths across the six primary sites. The early-period colorectal-by-brain coefficient was −1.006 (SE 0.629). Across the exploratory penalty grid, interaction coefficients ranged from −1.006 to 0.809 without penalization and from −0.406 to 0.247 at the largest penalty (**Supplementary Tables S6B, C**).

### 3.5 Sensitivity and focused interaction analyses

Schoenfeld-residual tests identified nonproportionality for age, liver involvement, selected primary-site indicators, and gynecologic-by-liver and pancreatic-by-liver terms (**Supplementary Table S6**). In the exploratory piecewise Cox analysis, allowing the six site-specific liver associations to differ before and after six months improved fit (likelihood-ratio χ²=21.96, 6 df, P = 0.00123; **Supplementary Table S7**). Genitourinary liver HRs were 1.99 (95% CI 1.59–2.50) before six months and 1.59 (1.27–1.98) thereafter. Pancreatic estimates were 1.79 (95% CI 0.87–3.68) and 3.71 (1.50–9.20), respectively. The piecewise model retained evidence of nonproportionality (global rank-Schoenfeld χ²=64.32, 32 df, P = 0.000604; **Supplementary Table S6D**).

The 12-month logistic analysis included 2,203 patients with known outcomes, including 1,676 deaths, and excluded 153 earlier-censored survivors. The global interaction P value was 0.00793 (**Supplementary Table S8**). Excluding eight stage-discordant cases left 2,348 patients and 2,080 deaths; the global interaction test yielded χ²=45.73 (20 df, P = 0.000877; **Supplementary Table S9**). Interaction P values were 0.00105 for cancer-specific death, 0.0181 for all malignant primaries, 0.00113 after excluding primary liver tumors, and 0.000723 after adding grade-supported code 8246 tumors.

The grade-supported 8246 analysis increased the cohort from 6,044 to 9,040 patients. The pancreatic-versus-genitourinary liver PR changed from 3.80 (95% CI 3.39–4.25) to 3.59 (95% CI 3.23–3.98), and the colorectal contrast changed from 2.88 (95% CI 2.56–3.23) to 2.90 (95% CI 2.62–3.21). Liver HRs were 1.70 (95% CI 1.45–1.99) for genitourinary, 2.02 (95% CI 1.54–2.67) for colorectal, 2.11 (95% CI 1.40–3.16) for pancreatic, and 1.02 (95% CI 0.77–1.36) for upper gastrointestinal primaries. The colorectal bone HR decreased from 1.36 to 1.18 (95% CI 0.99–1.41).

In the focused analysis, the liver-by-primary-site interaction had a likelihood-ratio χ² of 18.84 (5 df, P = 0.00205; **Figure 2B**). Adjusted liver HRs were 0.99 (95% CI 0.68–1.44) in upper gastrointestinal, 2.80 (95% CI 1.59–4.95) in pancreatic, 1.68 (95% CI 1.43–1.97) in genitourinary, and 2.48 (95% CI 1.71–3.62) in colorectal primaries. The 5-df interaction yielded χ²=12.99 (P = 0.0235) in 2010–2017 and χ²=15.61 (P = 0.00807) in 2018–2022. Pancreatic liver HRs were 3.75 (95% CI 1.73–8.14) and 3.23 (95% CI 1.16–9.02), respectively; upper gastrointestinal estimates remained near 1 in both periods.

### 3.6 Associations across calendar periods

Seven adjusted site-organ contrasts met FDR<0.05 during 2010–2017. All retained the same direction during 2018–2022, and all later-period 95% CIs excluded 1 (**Table 3**). The pancreatic-versus-genitourinary liver PR was 3.77 (95% CI 3.16–4.50) in 2010–2017 and 3.97 (95% CI 3.36–4.68) in 2018–2022. The corresponding colorectal PRs were 2.95 (95% CI 2.46–3.53) and 3.06 (95% CI 2.58–3.62).

**Table 3:** Temporal reassessment of discovery-defined associations within SEER.

Domain	Primary-site comparison or stratum	2010–2017 estimate (95% CI)	2018–2022 estimate (95% CI)
Bone metastatic prevalence	Gynecologic vs genitourinary	PR 0.56 (0.36–0.87)	PR 0.61 (0.41–0.91)
Bone metastatic prevalence	Colorectal vs genitourinary	PR 0.63 (0.45–0.90)	PR 0.55 (0.40–0.77)
Bone metastatic prevalence	Upper gastrointestinal vs genitourinary	PR 0.50 (0.33–0.74)	PR 0.60 (0.42–0.85)
Bone metastatic prevalence	Other vs genitourinary	PR 0.58 (0.43–0.79)	PR 0.52 (0.39–0.70)
Liver metastatic prevalence	Colorectal vs genitourinary	PR 2.95 (2.46–3.53)	PR 3.06 (2.58–3.62)
Liver metastatic prevalence	Pancreatic vs genitourinary	PR 3.77 (3.16–4.50)	PR 3.97 (3.36–4.68)
Liver metastatic prevalence	Upper gastrointestinal vs genitourinary	PR 2.19 (1.79–2.69)	PR 2.46 (2.03–2.99)
Liver prognosis	Genitourinary stratum	HR 1.83 (1.43–2.34)	HR 1.61 (1.26–2.05)
Liver prognosis	Pancreatic stratum	HR 4.10 (1.80–9.35)	HR 4.64 (1.44–14.91)
Bone prognosis	Other-site stratum	HR 1.82 (1.24–2.67)	HR 1.35 (0.93–1.96)
Lung prognosis	Other-site stratum	HR 1.82 (1.17–2.84)	HR 1.43 (0.86–2.35)

PRs were adjusted for age, sex, race/ethnicity, diagnosis year, and morphology. HRs were derived from the adjusted primary-site-by-organ Cox model. Associations were selected by FDR<0.05 in 2010–2017 and re-estimated in 2018–2022. HRs compare organ-positive with organ-negative patients within the specified primary-site stratum, adjusting for age, sex, race/ethnicity, diagnosis year, morphology, primary site, the four organ indicators, and their site interactions. PR, prevalence ratio; HR, hazard ratio; CI, confidence interval; FDR, false discovery rate.

Four primary-site-specific organ survival associations met the discovery FDR criterion. All retained their direction in 2018–2022, but only two later-period CIs excluded 1 (**Table 3**). For liver metastasis, genitourinary HRs were 1.83 (95% CI 1.43–2.34) and 1.61 (95% CI 1.26–2.05), and pancreatic HRs were 4.10 (95% CI 1.80–9.35) and 4.64 (95% CI 1.44–14.91). In the statistically aggregated other-site group, bone HRs were 1.82 (95% CI 1.24–2.67) and 1.35 (95% CI 0.93–1.96), whereas lung HRs were 1.82 (95% CI 1.17–2.84) and 1.43 (95% CI 0.86–2.35). The 20-df global interaction test yielded P = 0.052 and P = 0.086 in the two periods, whereas the focused 5-df liver interaction yielded P = 0.0235 and P = 0.00807.

### 3.7 Full interactions and temporal predictive value

Adding the full interaction matrix changed the 2018–2022 C-index from 0.59364 to 0.59532 (Δ=0.00169) in the adjusted survival analysis (**Figure 3**). In the 12-month mortality analysis, the later-period AUC changed from 0.6315 to 0.6184 (Δ=−0.0131). Across the three case-definition sensitivity analyses, changes in C-index ranged from −0.00318 to 0.00262.

**Figure 3:**
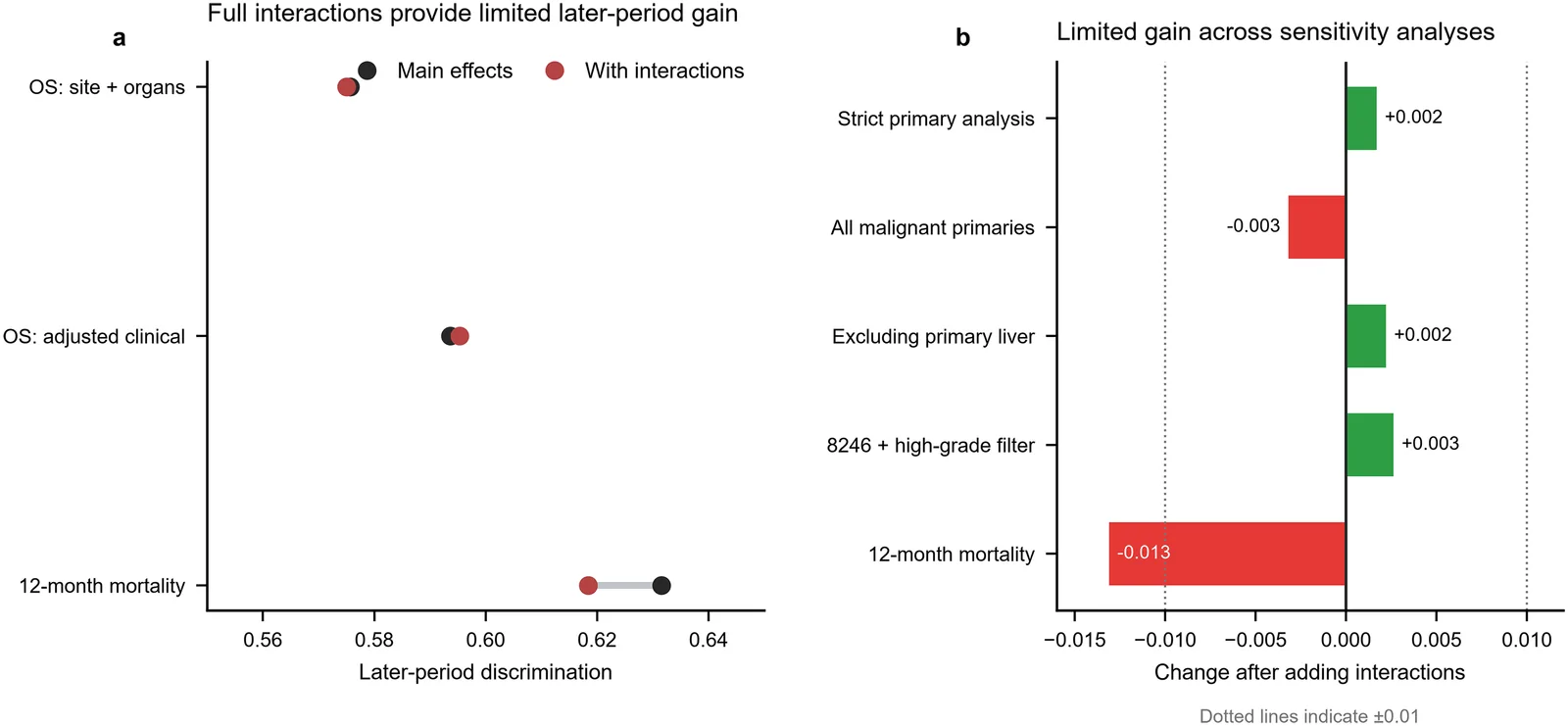
Limited incremental predictive value of full primary-site-by-organ interactions in temporal assessment. **(A)** Discrimination in the 2018–2022 later-period cohort for models trained using 2010–2017 diagnoses. Black markers show main-effects models and red markers show models additionally containing primary-site-by-organ interactions. **(B)** Change in later-period discrimination after adding interactions in the strict primary analysis and sensitivity analyses. Survival models use Harrell C-index and the 12-month binary-outcome model uses AUC. Dotted lines denote changes of ±0.01 as visual reference values, not clinical-use thresholds. The strict adjusted survival model gains 0.00169 in C-index, whereas the 12-month model loses 0.0131 in AUC. AUC, area under the receiver-operating-characteristic curve; OS, overall survival.

### 3.8 Development and temporal assessment of the parsimonious model

Discrimination increased and 12-month Brier scores decreased across M0, M1, M2, and M3 (**Table 4**; **Figure 4**). In later-period assessment, M0, M1, M2, and M3 yielded Harrell C-indices of 0.545, 0.573, 0.598, and 0.607, respectively. Corresponding 12-month AUCs were 0.550, 0.614, 0.660, and 0.672, and 12-month Brier scores were 0.212, 0.206, 0.198, and 0.196. Relative to M1, M3 increased the C-index by 0.0332 and reduced the 12-month Brier score by 0.0102. Relative to M2, paired bootstrap differences were 0.0085 (95% CI 0.0035–0.0139) for the C-index, 0.0119 (95% CI 0.0033–0.0231) for 12-month AUC, and −0.0020 (95% CI −0.0038 to −0.0004) for 12-month Brier score (**Supplementary Table S10**). The C-index and Brier-score improvements of M3 over M1 met the protocol criteria for subsequent risk-group and subgroup analyses.

**Table 4:** Performance of prespecified prognostic models in the 2018–2022 later-period cohort.

Model	Predictor set	Harrell C-index	12-month AUC	12-month Brier score	12-month calibration slope	12-month CITL
M0 Basic	Age spline, sex, morphology	0.545 (0.529–0.562)	0.550 (0.521–0.584)	0.212 (0.201–0.221)	0.591	−0.029
M1 Site	M0 + primary site	0.573 (0.556–0.590)	0.614 (0.587–0.643)	0.206 (0.196–0.216)	0.859	−0.044
M2 Four-organ count	M1 + number of involved recorded organs	0.598 (0.579–0.617)	0.660 (0.630–0.692)	0.198 (0.188–0.209)	0.839	−0.082
M3 Spectrum	M1 + bone, brain, liver, lung indicators	0.607 (0.588–0.624)	0.672 (0.639–0.706)	0.196 (0.185–0.207)	0.911	−0.090

Values in parentheses are 95% percentile confidence intervals from 300 patient-level bootstrap resamples of the later-period cohort, with the fitted penalized models and penalties fixed; these intervals do not include model-selection uncertainty. Calibration slope and CITL are point estimates. The M2 count was encoded as 0, 1, 2, or ≥3 organs. AUC, cumulative/dynamic area under the curve; CITL, calibration-in-the-large. Lower Brier scores indicate lower prediction error.

**Figure 4:**
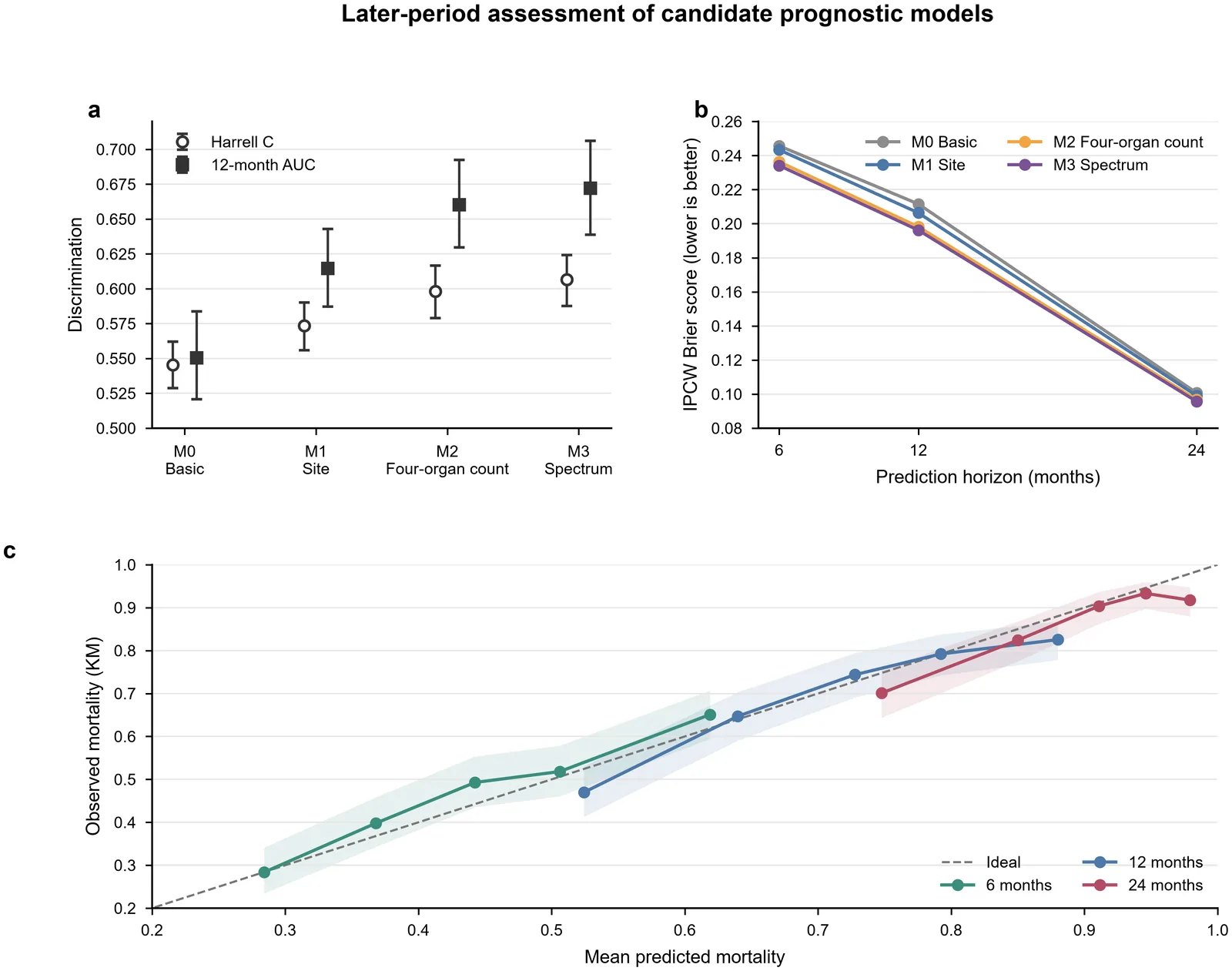
Temporal assessment of candidate prognostic models. **(A)** Harrell C-index and 12-month cumulative/dynamic AUC for M0–M3 in the 2018–2022 later-period cohort; bars show 95% CIs from 300 patient-level bootstrap resamples. **(B)** IPCW Brier scores at 6, 12, and 24 months. **(C)** Calibration of M3 at 6, 12, and 24 months; points compare mean predicted mortality with Kaplan–Meier-observed mortality in fifths of predicted risk. Shaded bands connect pointwise 95% confidence limits for Kaplan–Meier-observed mortality within each risk group; the dashed diagonal indicates perfect agreement. M0 includes age spline, sex, and morphology; M1 adds primary site; M2 adds grouped four-organ count to M1; M3 adds four organ indicators to M1. AUC, area under the curve; CI, confidence interval; IPCW, inverse probability of censoring weighting; KM, Kaplan–Meier.

For M3, the later-period C-index was 0.607 (95% CI 0.588–0.624). Time-dependent AUCs at 6, 12, and 24 months were 0.646, 0.672 (95% CI 0.639–0.706), and 0.685; Brier scores were 0.234, 0.196 (95% CI 0.185–0.207), and 0.096. Calibration slopes were 1.055, 0.911, and 0.670 at the same horizons. At 12 months, mean predicted mortality was 0.713 versus an observed risk of 0.696; at 24 months, the corresponding values were 0.887 and 0.856, indicating increasing overprediction at the later horizon. The development C-index was 0.623; bootstrap optimism was 0.0058, giving an optimism-corrected estimate of 0.617. The selected ridge penalty strength was λ=39.81. An elastic-net sensitivity retained 14 of 15 coefficients and yielded C-index 0.6062, 12-month AUC 0.6718, and Brier score 0.1964 (**Supplementary Table S11**). The fitted age effect and penalized categorical coefficients are shown in **Supplementary Figure 3**. Relative to its unpenalized comparator, M3 reduced the standardized coefficient norm by 4.94% and the development linear-predictor standard deviation by 3.30%. Ten of its 11 nonspline coefficient magnitudes decreased, whereas the Other-site coefficient magnitude increased by 24.2%; none changed sign (**Supplementary Tables S11B, C**). The unpenalized M3 analogue showed nonproportionality (global rank-Schoenfeld χ²=54.28, 14 df, P = 1.15×10^-6^; **Supplementary Table S6D**).

Performance was similar in 2018–2019 and 2020–2022 for discrimination (C-index 0.616 vs 0.603; 12-month AUC 0.675 vs 0.674), but 12-month Brier score was higher during 2020–2022 (0.202 vs 0.186; **Supplementary Table S12**). Mean predicted 12-month mortality exceeded Kaplan–Meier-observed mortality in 2020–2022 (0.715 vs 0.677) whereas the model slightly underpredicted in 2018–2019 (0.709 vs 0.725). Chemotherapy recording was stable across 2010–2017, 2018–2019, and 2020–2022 (68.2%, 68.5%, and 69.0%), while recorded radiotherapy decreased (24.6%, 23.5%, and 20.1%) and surgery was less frequent after 2017 (35.3%, 29.3%, and 30.2%; **Supplementary Table S13**).

### 3.9 Risk groups and subgroup performance

Development-set tertiles of predicted 12-month mortality (0.641 and 0.775) separated the later-period cohort into low- (n=426), intermediate- (n=469), and high-risk (n=479) groups (**Figure 5A**). Median OS was 10.9, 6.8, and 4.3 months, respectively (**Figure 5B**); 12-month OS was 47.2%, 28.2%, and 17.7%. The survival curves differed (log-rank χ²=111.25, P = 6.95×10^-^²^5^).

**Figure 5:**
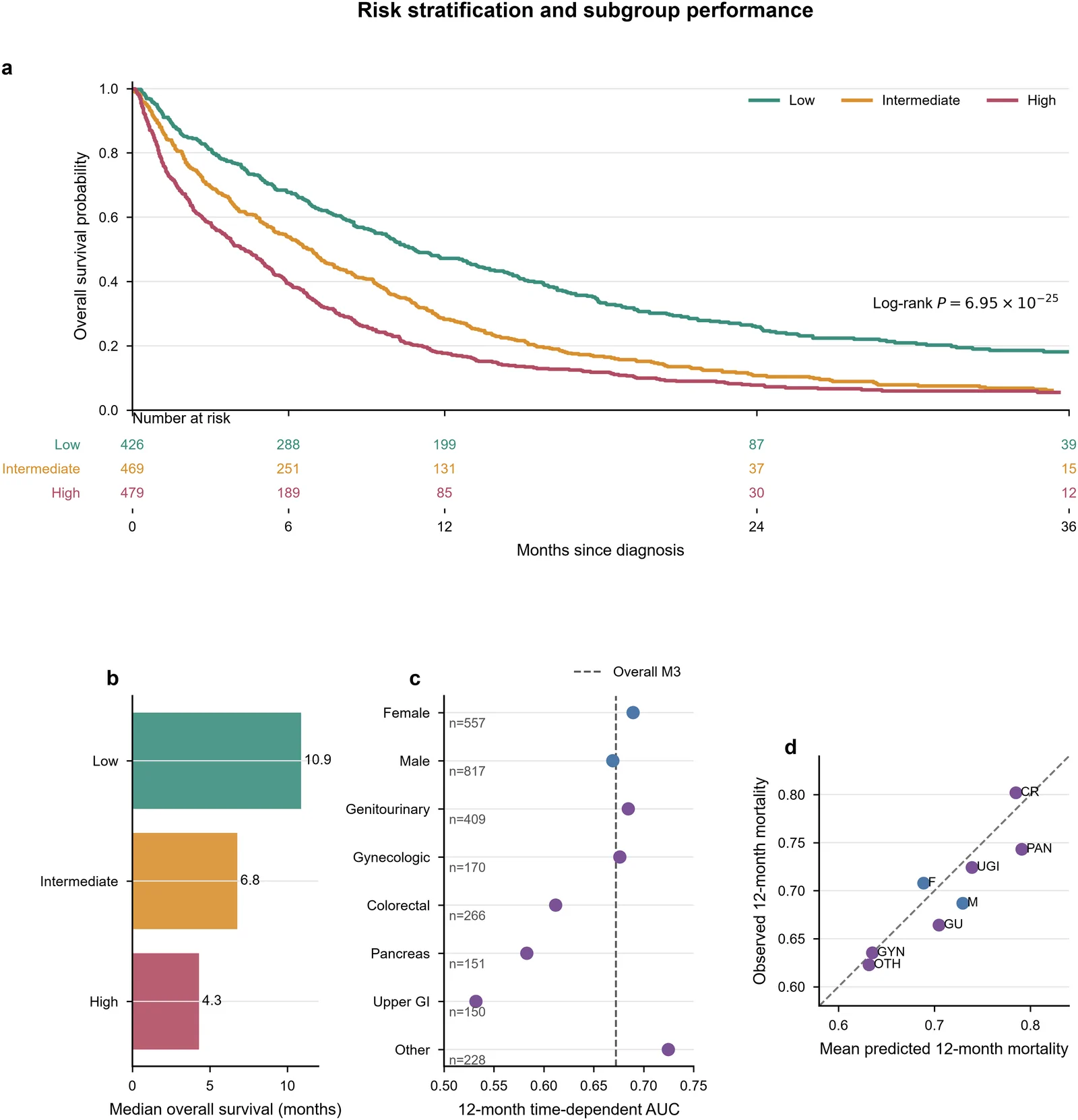
Risk stratification and subgroup performance. **(A)** Kaplan–Meier OS in the later-period cohort after applying unchanged development-cohort tertile cut points for predicted 12-month mortality (0.641 and 0.775). **(B)** Median OS for low-, intermediate-, and high-risk groups. **(C)** M3 12-month time-dependent AUC by sex and primary site. **(D)** Mean predicted versus Kaplan–Meier-observed 12-month mortality in the same subgroups. Subgroup estimates are descriptive and were not multiplicity adjusted. The dashed vertical line in C marks overall M3 AUC; the dashed diagonal in D marks perfect agreement. Panels B–D show point estimates without confidence intervals. OS, overall survival; AUC, area under the curve; F, female; M, male; GU, genitourinary; GYN, gynecologic; CR, colorectal; PAN, pancreatic; UGI, upper gastrointestinal; OTH, other extrapulmonary sites.

Subgroup discrimination and mean-risk calibration are shown in **Figures 5C, D**. The 12-month AUC was 0.689 in females and 0.669 in males. Across primary sites, AUC ranged from 0.532 in upper gastrointestinal to 0.725 in other primaries; values were 0.685 for genitourinary, 0.676 for gynecologic, 0.612 for colorectal, and 0.583 for pancreatic primaries. The upper gastrointestinal calibration slope was approximately 0.13.

## 4 Discussion

### 4.1 Primary-site-specific metastatic heterogeneity

Primary-site differences in recorded metastatic patterns were more pronounced than their contribution to survival prediction. Liver involvement varied substantially across primary sites, and selected organ-specific survival associations also differed. However, site-by-organ interactions contributed little to predictive performance in the later-period cohort. Most of the predictive gain from metastatic variables was captured by the recorded four-organ count.

The predominance of liver involvement in gastrointestinal and pancreatic primaries may partly reflect portal drainage, while molecular differences among NECs could also contribute (9, 10, 22). Recorded distributions also depend on imaging selection, staging practice, and documentation. These factors provide alternative explanations for the observed patterns, alongside the biological processes described by the seed-and-soil framework. Prospective studies combining standardized staging with pathological and molecular characterization could help distinguish their contributions.

The near-null average liver association in upper gastrointestinal primaries may conceal differences over follow-up. The piecewise estimates differed between intervals, with substantial uncertainty. Case mix, restriction to patients with recorded metastases, and adjustment for co-metastases may also influence this association. The available data cannot distinguish these explanations. Time variation in liver associations limits interpretation of a single Cox HR as a constant association throughout follow-up.

Previous population-based work reported that extrapulmonary NEC incidence, stage distribution, and survival differ by anatomical site (8). Clinical studies emphasize that well-differentiated grade 3 neuroendocrine tumors and poorly differentiated NEC are distinct entities despite overlap in high proliferative activity (2, 4). The present findings add synchronous recorded metastatic distribution to this recognized anatomical heterogeneity. They also indicate that variation in organ-specific prognostic associations need not improve prediction in later patients.

### 4.2 From metastatic heterogeneity to parsimonious prediction

Differences in survival associations across primary sites need not improve prediction for individual patients. Although the pooled interaction test supported heterogeneity, the full interaction model added little predictive information in the later cohort. Sparse site–organ strata may limit the precision of interaction estimates and their contribution to prediction in later patients. The small predictive gain therefore does not imply that organ-specific survival associations are identical across primary sites. The small brain-positive strata and penalty-sensitive interaction estimates further limit conclusions about the absence of effect modification.

Within this dataset, the recorded four-organ count accounted for most of the predictive gain from metastatic variables beyond baseline characteristics and primary site. Individual organ indicators provided a small additional contribution, suggesting substantial overlap between information on the extent and location of recorded organ involvement.

Overall discrimination remained modest, with small gains from organ identity beyond the recorded four-organ count. Calibration was closer at 6 and 12 months than at 24 months. Changes in risk-set composition and subsequent care may contribute to this pattern, although their contributions could not be separated. Performance also varied across primary sites, particularly in upper gastrointestinal tumors, indicating that aggregate estimates can conceal subgroup differences. These findings limit the precision of individual risk estimates and their application to clinical decisions (19–21).

Both cohorts came from SEER, leaving performance in other populations uncertain. Selection of M3 also used later-period performance, which may have favored its reported estimates. The later cohort therefore provided temporal assessment with some model-selection dependence (19–21). Differences in prediction error and calibration between 2018–2019 and 2020–2022 may reflect changes in case mix, ascertainment, or care. The period comparisons do not isolate the contribution of the pandemic.

### 4.3 Clinical implications, strengths, and limitations

The site-specific prevalence patterns support further investigation of differences in metastatic presentation across EP-PD-NEC primary sites. Standardized documentation of imaging and staging would help determine how much of this variation reflects disease distribution versus clinical ascertainment. SEER lacked imaging modality, assessment intensity, and the indications for testing, limiting inferences about site-specific assessment strategies.

The prognostic heterogeneity observed here complements the clinical heterogeneity described in EP-PD-NEC. Evidence remains limited for local treatment outside selected localized disease and for later-line systemic therapies (7). Understanding whether metastatic patterns modify treatment benefit would require treatment-specific comparative studies.

The population-based cohort allowed metastatic patterns to be compared across several uncommon primary sites within a common data source. The strict morphology definition reduced diagnostic heterogeneity, while organ-specific denominators retained patients with known status for each organ. Evaluation across nonoverlapping periods provided evidence on the persistence of the observed patterns over time.

Several limitations arise from registry coverage. SEER records bone, brain, liver, and lung involvement only at diagnosis. Peritoneal, distant nodal, adrenal, other organ, and later metastases are incompletely represented. Four negative organ indicators therefore do not imply an absence of distant disease, and variation in detection intensity may contribute to the observed prevalence patterns. Missing four-organ status affected 3.7% of patients and was associated with age, diagnosis year, and mortality. Although unknown-as-negative and multiple-imputation analyses yielded similar main PRs, missingness may remain informative. Survival and prediction analyses required complete four-organ status and remained susceptible to selection related to missingness. Crude death proportions in the missingness comparison are also affected by differences in diagnosis year and follow-up. Moreover, the registry identifier needed for cluster-robust inference was unavailable in the standard research export used for this study; individual-level robust standard errors may therefore understate uncertainty if meaningful within-registry correlation remains. Stable imputation diagnostics do not establish that missingness was at random or exclude bias from differential staging.

Pathological classification was based on registry coding without central review, Ki-67 index, mitotic count, necrosis assessment, differentiation review, immunohistochemistry, or molecular data. The absence of central pathology may also permit site-dependent misclassification across primary-site groups. Excluding code 8246 favored specificity but may have omitted true PD-NEC, whereas including it without grade support could mix well-differentiated grade 3 tumors with NEC. The broader grade-supported analysis preserved major liver associations while attenuating the colorectal bone estimate. The residual other-site group combined biologically diverse tumors because finer divisions produced sparse interaction cells; its pooled estimates may obscure differences between constituent sites.

Clinical information was limited, including performance status, laboratory values, quantitative tumor burden, systemic regimens, response, recurrence, and imaging practice. Residual differences in these factors may influence both survival associations and prediction. Nonproportional liver associations mean that the reported Cox HRs summarize associations that varied over follow-up. Sparse brain-metastasis and four-organ strata limited precision, particularly within individual primary-site groups. Excluding early-censored survivors from the 12-month logistic analysis may also introduce selection bias if their outcomes differed from those with known 12-month status.

The piecewise extension did not remove all nonproportionality, and unpenalized analogues of the prediction models also showed time variation. Ridge shrinkage stabilizes coefficients but does not correct this assumption; fixed-horizon performance therefore remains important, and a single HR should not be interpreted as constant over follow-up.

## 5 Conclusion

Primary site was associated with recorded metastatic patterns at diagnosis in EP-PD-NEC. The recorded four-organ count accounted for most of the predictive gain from metastatic variables. Organ identity added a small improvement, whereas site-by-organ interactions contributed little to later-period prediction. Overall discrimination remained modest.

## Data Availability

Publicly available datasets were analyzed in this study. This data can be found here: The individual-level SEER data analyzed in this study are available to registered users from the National Cancer Institute SEER Program (https://seer.cancer.gov/data/) under a data-use agreement and the terms applicable to the selected SEER database. The authors are not permitted to redistribute raw or derived patient-level SEER data. The statistical analysis code is publicly available at https://github.com/wusmall1994-create/seer-ep-pd-nec-analysis-code and is archived in Zenodo as version 1.0.0 (https://doi.org/10.5281/zenodo.22057954). Additional revision analyses are provided as **Supplementary Analysis Code**. The cited v1.0.0 archive represents the original pipeline and does not include all subsequent revision analyses.
