# Supplementary material for: Primary-site variation in recorded metastatic patterns and survival associations in extrapulmonary poorly differentiated neuroendocrine carcinoma
Source: Front Endocrinol (Lausanne). 2026 Oct 22;17:1975853. doi: 10.3389/fendo.2026.1975853 (PMC13735552; doi:10.3389/fendo.2026.1975853)
Supplement: Supplementary file 2 [file SupplementaryFile1.docx]

**Supplementary Material**

Primary-site variation in recorded metastatic patterns and survival associations in extrapulmonary poorly differentiated neuroendocrine carcinoma

# Supplementary Methods

Cohort and variables. Strict EP-PD-NEC used ICD-O-3 morphology codes 8013 and 8041–8045 and excluded pulmonary primaries. The analysis began in 2010 because bone, brain, liver, and lung metastasis-at-diagnosis variables were available for the complete study interval. The residual Other category was created only to stabilize sparse estimates.

Missing-status analyses. The primary prevalence analysis excluded unknown status only for the organ being modeled. We additionally compared complete and incomplete four-organ records; repeated modified Poisson models after recoding unknown values as negative; and performed 20 chained-equation imputations using age, sex, diagnosis year, morphology, race/ethnicity, primary site, stage, and the other organ indicators. Coefficients and variances were combined with Rubin's rules. Here complete case denotes known status for the target organ, not necessarily a complete four-organ panel. Each imputation used 10 chained updates of binary logistic models and random seed 20260927. Missing indicators were initialized by site-specific Bernoulli draws; model coefficients were drawn from their estimated multivariate normal distribution before Bernoulli imputation. Other organs and the listed covariates entered each conditional model. Poisson coefficients and HC0 covariance matrices were combined on the log scale using Rubin within- and between-imputation variance; reported intervals used a normal approximation. A separate diagnostic run used 20 chains with seeds 20260929–20260948 and 50 updates per chain. Continuous imputation predictors were standardized for numerical conditioning. Split-chain R-hat was calculated for each imputed organ frequency using iterations 11–50, split into two 20-update blocks per chain. Pooled estimates at updates 10 and 50 were compared; Monte Carlo SE of a pooled log-PR was estimated as sqrt(B/20), where B is the between-imputation variance, and compared with its total SE. These summary diagnostics do not establish convergence of every parameter or the missing-at-random assumption. The original 20-imputation estimates were retained. These analyses address prevalence estimates only.

Prediction comparisons. M0 contained age spline, sex, and morphology; M1 added primary site; M2 added the number of involved recorded organs among bone, brain, liver, and lung; M3 replaced that count with four organ indicators. Ridge penalties were selected by five-fold cross-validation. M3 used penalty strength λ=39.81, implemented as the alpha parameter in scikit-survival CoxPHSurvivalAnalysis. Later-period performance intervals used fixed-model bootstrap resampling without refitting or retuning. A separate 500-resample paired bootstrap used identical later-period patient indices for M2 and M3. Point differences are evaluated in the original later-period sample, with percentile intervals from the paired bootstrap distribution. M2 and M3 are alternative, rather than mutually nested, representations.

Shrinkage comparison. Each M0–M3 fit was compared with an unpenalized Cox model using the same cohort, knot locations, scaling, and Breslow tie handling. The centered natural-spline design has one linear dependency: M0–M3 have 6, 11, 14, and 15 columns but ranks 5, 10, 13, and 14. One redundant spline column was omitted for the unpenalized fit, and its linear predictor was mapped back to the minimum-norm representation in the original standardized basis. Norm reduction was 100×(1−||βpen||/||βunpen||); linear-predictor SD reduction used the corresponding SD ratio in the development data. Coordinate reduction was 100×(1−|βpen|/|βunpen|); negative values indicate expansion. Ratios were omitted for |βunpen|≤0.01. Spline coordinates and their shrinkage depend on the basis and should not be interpreted separately as age effects. Design rank is not the effective degrees of freedom of a ridge fit. The existing penalty selection and predictions were unchanged.

Pandemic-period assessment. The unchanged M3 model was evaluated separately in 2018–2019 and 2020–2022. Treatment fields were summarized descriptively and were not entered into the prognostic model because treatment choice is postbaseline, coarsely measured, and confounded by indication. Observed 12-month mortality was estimated as one minus Kaplan–Meier survival; two patients in 2018–2019 and four in 2020–2022 were censored before 12 months.

Registry clustering. The standard SEER Research case-listing export available to the authors did not contain a registry identifier. Modified Poisson models therefore used individual-level heteroscedasticity-consistent standard errors; registry-clustered inference could not be performed.

Prediction implementation. Age used a natural cubic spline with boundary knots 40 and 85 and interior knots 60 and 70.4 years. The design columns were standardized using development means and standard deviations. A 26-value logarithmic penalty grid from 10^-6 to 10^4 was searched with five-fold cross-validation, minimizing average IPCW Brier score at 6, 12, and 24 months. The original implementation formed spline and scaling transformations on the development cohort before cross-validation; this and fixed-penalty optimism estimation do not fully repeat the entire tuning pipeline.

Censoring and calibration. Time-dependent AUC and Brier score used scikit-survival censoring estimates from the development outcomes. Calibration used reverse Kaplan–Meier censoring survival within the evaluated cohort: deaths by the horizon received weight 1/G(T-), survivors beyond the horizon 1/G(t-), and earlier-censored observations zero weight. G was bounded below by 10^-4. Predicted risk was bounded to [10^-5, 1-10^-5]; weighted logistic regression with logit predicted risk as an offset estimated calibration-in-the-large, while an intercept and fitted logit coefficient estimated the calibration slope. Grouped calibration used Kaplan–Meier risk within predicted-risk fifths.

Model advancement. The original protocol compared M3 with M1 in the later cohort, not with M2. Its non-DCA criteria were a Harrell/Uno C gain of at least 0.01, 12-month Brier reduction of at least 0.005, or absolute mean-risk calibration error reduction of at least 0.02 at two horizons, without C loss above 0.01 or Brier increase above 0.005. The implemented calibration criterion used absolute mean-risk error, although the protocol called it calibration-in-the-large error. M3 met the discrimination (+0.03316) and Brier (0.01017) criteria, but not the calibration criterion. The historical gate also included a DCA branch; that branch is not used in the revised interpretation and its removal does not change the advancement decision. Later-period data therefore contributed to advancement as well as assessment. These are reporting criteria, not clinical-use thresholds.

Additional survival analyses. The binary 12-month analysis used 2,203 known outcomes and excluded 153 survivors censored before 12 months. Site-specific liver odds ratios were formed from main and interaction coefficients and their full covariance matrix; odds ratios are not hazard ratios. The exploratory piecewise Cox sensitivity split follow-up at six months, allowed separate interval baseline hazards, retained age, year, sex, race, morphology, primary site and all four organ indicators, and included site-by-liver effects. Late-interval interactions were added for age and gynecologic, pancreatic and other primary sites, and for each of the six site-specific liver effects. A six-df likelihood-ratio test compared models with and without the liver time terms. The six-month cut was chosen for this sensitivity analysis and is not claimed to be an a priori biological threshold. Excluding stage-discordant cases repeated the full 20-df interaction comparison. These additional checks were implemented in R version 4.6.1 using survival version 3.8.6; the original analyses used Python version 3.12.14.

Sparse interactions and residual diagnostics. The full interaction model has 17 main-effect and 20 interaction coefficients. Positive and negative organ-specific counts and deaths were tabulated within each site and period; these overlapping counts are not independent effective sample sizes. Interaction coefficients were refitted with lifelines CoxPHFitter at penalizer values 0, 0.001, 0.01, 0.1, and 1, using Efron ties and penalizing all coefficients. This exploratory diagnostic grid was not tuned for performance and did not replace the original unpenalized interaction tests. The lifelines normalized, sample-size-scaled penalty is not numerically interchangeable with scikit-survival alpha. Rank-transformed Schoenfeld tests in R evaluated the original and piecewise models and identifiable unpenalized M0–M3 analogues; the latter used Breslow ties to match prediction fitting. Global and term-level diagnostics are reported in Table S6D. These analogue tests identify concerns for the predictor specification but are not formal PH tests of the ridge estimator. Diagnostic Python packages were numpy 2.3.5, pandas 2.3.3, scipy 1.18.1, patsy 1.0.3, statsmodels 0.15.0, lifelines 0.30.3, scikit-learn 1.9.1, and scikit-survival 0.28.0.

Grade-supported definition. Code 8246 was included only with old grade III/IV or a newer grade label indicating poorly differentiated, high grade, undifferentiated, or the coded categories (3), (4), or (5) used by the original extraction script. This operational registry rule is not a centrally reviewed pathological diagnosis and may behave differently across sites and coding periods.

## Analysis populations

| **Population** | **Definition** | **n** |
| --- | --- | --- |
| Strict eligible cohort | First malignant, microscopically confirmed nonpulmonary NEC; valid survival | 6,044 |
| Complete four-organ cohort | Known bone, brain, liver, and lung status | 5,820 |
| Metastatic-spectrum cohort | Complete status and at least one of four organs positive | 2,356 (2,087 deaths) |
| Prediction cohort | Distant stage, complete status, valid OS | 3,018 |
| Development cohort | Prediction cohort, 2010–2017 | 1,297 (1,241 deaths) |
| Later-period cohort | Prediction cohort, 2018–2022 | 1,374 (1,214 deaths) |

# Supplementary Table S1. ICD-O-3 topography mapping to the six primary-site groups

| **Six-group category** | **ICD-O-3 topography codes or component categories** | **Operational mapping** |
| --- | --- | --- |
| Genitourinary | C60.0–C68.9 | Genitourinary |
| Gynecologic | C51.0–C58.9 | Gynecologic |
| Colorectal | C18.0 and C18.2–C18.9; C19.0–C21.9 | Colon plus rectum/anus; C18.1 grouped with appendix |
| Pancreatic | C25.0–C25.9 | Pancreas |
| Upper gastrointestinal | C15.0–C16.9 | Esophagus plus stomach |
| Other | C00.0–C14.9; C17.0–C17.9; C18.1; C22.0–C24.9; C30.0–C32.9; C50.0–C50.9; all remaining known nonpulmonary sites not assigned above | Head and neck, small bowel/appendix, hepatobiliary, breast, and residual uncommon sites |

# Supplementary Table S2. Components of the statistical Other category

| **other_component** | **organ** | **all_n** | **known_n** | **positive_n** | **prevalence_pct** |
| --- | --- | --- | --- | --- | --- |
| Breast | bone | 125 | 120 | 19 | 15.83 |
| Breast | brain | 125 | 119 | 3 | 2.521 |
| Breast | liver | 125 | 120 | 18 | 15 |
| Breast | lung | 125 | 117 | 10 | 8.547 |
| Head and neck | bone | 431 | 424 | 46 | 10.85 |
| Head and neck | brain | 431 | 425 | 10 | 2.353 |
| Head and neck | liver | 431 | 425 | 48 | 11.29 |
| Head and neck | lung | 431 | 425 | 25 | 5.882 |
| Hepatobiliary | bone | 294 | 286 | 25 | 8.741 |
| Hepatobiliary | brain | 294 | 285 | 2 | 0.7018 |
| Hepatobiliary | liver | 294 | 285 | 85 | 29.82 |
| Hepatobiliary | lung | 294 | 283 | 19 | 6.714 |
| Other | bone | 154 | 144 | 27 | 18.75 |
| Other | brain | 154 | 146 | 8 | 5.479 |
| Other | liver | 154 | 149 | 70 | 46.98 |
| Other | lung | 154 | 144 | 18 | 12.5 |
| Small bowel/appendix | bone | 92 | 91 | 2 | 2.198 |
| Small bowel/appendix | brain | 92 | 91 | 1 | 1.099 |
| Small bowel/appendix | liver | 92 | 92 | 41 | 44.57 |
| Small bowel/appendix | lung | 92 | 91 | 5 | 5.495 |

All N is the component population; known N is the organ-specific denominator after excluding unknown status. Prevalence is positive N / known N × 100%.

The Other category is a statistical aggregation. Component differences preclude a single biological interpretation.

# Supplementary Table S3. Missing-status frequency by primary site

| Primary site | N | Any unknown n (%) | Bone unknown n (%) | Brain unknown n (%) | Liver unknown n (%) | Lung unknown n (%) |
| --- | --- | --- | --- | --- | --- | --- |
| Genitourinary | 2131 | 70 (3.3) | 42 (2.0) | 49 (2.3) | 40 (1.9) | 55 (2.6) |
| Gynecologic | 920 | 28 (3.0) | 17 (1.8) | 19 (2.1) | 18 (2.0) | 22 (2.4) |
| Colorectal | 908 | 33 (3.6) | 24 (2.6) | 21 (2.3) | 16 (1.8) | 25 (2.8) |
| Pancreas | 410 | 21 (5.1) | 16 (3.9) | 13 (3.2) | 9 (2.2) | 14 (3.4) |
| Upper GI | 579 | 30 (5.2) | 20 (3.5) | 23 (4.0) | 16 (2.8) | 22 (3.8) |
| Other | 1096 | 42 (3.8) | 31 (2.8) | 30 (2.7) | 25 (2.3) | 36 (3.3) |
| Overall | 6044 | 224 (3.7) | 150 (2.5) | 155 (2.6) | 124 (2.1) | 174 (2.9) |

# Supplementary Table S4. Complete versus incomplete four-organ status

| Variable | Complete N | Complete value | Incomplete N | Incomplete value | Std. difference |
| --- | --- | --- | --- | --- | --- |
| Age, years | 5820 | 65.25 | 224 | 67.49 | -0.1569 |
| Diagnosis year | 5820 | 2017.54 | 224 | 2015.64 | 0.4889 |
| Male | 5820 | 0.5701 | 224 | 0.5804 | -0.02072 |
| Large-cell morphology | 5820 | 0.2019 | 224 | 0.192 | 0.02493 |
| Distant stage | 5820 | 0.5186 | 224 | 0.5491 | -0.0612 |
| Death | 5820 | 0.738 | 224 | 0.9152 | -0.4811 |
| Primary site: Genitourinary | 5820 | 0.3541 | 224 | 0.3125 | 0.08829 |
| Primary site: Gynecologic | 5820 | 0.1533 | 224 | 0.125 | 0.08165 |
| Primary site: Colorectal | 5820 | 0.1503 | 224 | 0.1473 | 0.008481 |
| Primary site: Pancreas | 5820 | 0.06684 | 224 | 0.09375 | -0.09902 |
| Primary site: Upper GI | 5820 | 0.09433 | 224 | 0.1339 | -0.1246 |
| Primary site: Other | 5820 | 0.1811 | 224 | 0.1875 | -0.01649 |

Values are means for age and diagnosis year and proportions for binary indicators. Standardized differences compare complete with incomplete records; positive values indicate higher values in complete records.

# Supplementary Table S5. Missing-status sensitivity and imputation diagnostics

| **organ** | **comparison** | **strategy** | **pr** | **ci_low** | **ci_high** |
| --- | --- | --- | --- | --- | --- |
| bone | Gynecologic vs Genitourinary | complete_case | 0.6413 | 0.4874 | 0.8436 |
| bone | Colorectal vs Genitourinary | complete_case | 0.5826 | 0.4651 | 0.7296 |
| bone | Pancreas vs Genitourinary | complete_case | 0.6478 | 0.4924 | 0.8522 |
| bone | Upper GI vs Genitourinary | complete_case | 0.5187 | 0.4032 | 0.6672 |
| bone | Other vs Genitourinary | complete_case | 0.5552 | 0.4564 | 0.6755 |
| bone | Gynecologic vs Genitourinary | unknown_negative | 0.6327 | 0.4805 | 0.8333 |
| bone | Colorectal vs Genitourinary | unknown_negative | 0.5744 | 0.4585 | 0.7196 |
| bone | Pancreas vs Genitourinary | unknown_negative | 0.6298 | 0.4785 | 0.829 |
| bone | Upper GI vs Genitourinary | unknown_negative | 0.5093 | 0.3958 | 0.6555 |
| bone | Other vs Genitourinary | unknown_negative | 0.5494 | 0.4512 | 0.669 |
| brain | Gynecologic vs Genitourinary | complete_case | 0.8048 | 0.4088 | 1.585 |
| brain | Colorectal vs Genitourinary | complete_case | 0.9779 | 0.5589 | 1.711 |
| brain | Pancreas vs Genitourinary | complete_case | 2.857 | 1.751 | 4.661 |
| brain | Upper GI vs Genitourinary | complete_case | 1.476 | 0.861 | 2.53 |
| brain | Other vs Genitourinary | complete_case | 0.9836 | 0.6002 | 1.612 |
| brain | Gynecologic vs Genitourinary | unknown_negative | 0.7997 | 0.4056 | 1.577 |
| brain | Colorectal vs Genitourinary | unknown_negative | 0.9747 | 0.5572 | 1.705 |
| brain | Pancreas vs Genitourinary | unknown_negative | 2.828 | 1.731 | 4.618 |
| brain | Upper GI vs Genitourinary | unknown_negative | 1.448 | 0.8434 | 2.488 |
| brain | Other vs Genitourinary | unknown_negative | 0.9772 | 0.596 | 1.602 |
| liver | Gynecologic vs Genitourinary | complete_case | 0.9649 | 0.7985 | 1.166 |
| liver | Colorectal vs Genitourinary | complete_case | 2.873 | 2.559 | 3.225 |
| liver | Pancreas vs Genitourinary | complete_case | 3.798 | 3.392 | 4.253 |
| liver | Upper GI vs Genitourinary | complete_case | 2.311 | 2.03 | 2.631 |
| liver | Other vs Genitourinary | complete_case | 1.309 | 1.138 | 1.506 |
| liver | Gynecologic vs Genitourinary | unknown_negative | 0.9514 | 0.7868 | 1.15 |
| liver | Colorectal vs Genitourinary | unknown_negative | 2.859 | 2.545 | 3.211 |
| liver | Pancreas vs Genitourinary | unknown_negative | 3.767 | 3.361 | 4.222 |
| liver | Upper GI vs Genitourinary | unknown_negative | 2.282 | 2.003 | 2.6 |
| liver | Other vs Genitourinary | unknown_negative | 1.297 | 1.126 | 1.493 |
| lung | Gynecologic vs Genitourinary | complete_case | 1.312 | 0.9938 | 1.733 |
| lung | Colorectal vs Genitourinary | complete_case | 1.253 | 0.9831 | 1.598 |
| lung | Pancreas vs Genitourinary | complete_case | 1.309 | 0.9725 | 1.762 |
| lung | Upper GI vs Genitourinary | complete_case | 1.035 | 0.7842 | 1.367 |
| lung | Other vs Genitourinary | complete_case | 0.7155 | 0.552 | 0.9274 |
| lung | Gynecologic vs Genitourinary | unknown_negative | 1.296 | 0.9802 | 1.713 |
| lung | Colorectal vs Genitourinary | unknown_negative | 1.24 | 0.9721 | 1.582 |
| lung | Pancreas vs Genitourinary | unknown_negative | 1.289 | 0.9571 | 1.735 |
| lung | Upper GI vs Genitourinary | unknown_negative | 1.019 | 0.7714 | 1.347 |
| lung | Other vs Genitourinary | unknown_negative | 0.7042 | 0.5427 | 0.9137 |
| bone | Gynecologic vs Genitourinary | multiple_imputation_m20 | 0.6297 | 0.4786 | 0.8285 |
| bone | Colorectal vs Genitourinary | multiple_imputation_m20 | 0.578 | 0.4615 | 0.724 |
| bone | Pancreas vs Genitourinary | multiple_imputation_m20 | 0.6465 | 0.4905 | 0.8521 |
| bone | Upper GI vs Genitourinary | multiple_imputation_m20 | 0.5158 | 0.4011 | 0.6632 |
| bone | Other vs Genitourinary | multiple_imputation_m20 | 0.553 | 0.4548 | 0.6724 |
| brain | Gynecologic vs Genitourinary | multiple_imputation_m20 | 0.7715 | 0.3923 | 1.517 |
| brain | Colorectal vs Genitourinary | multiple_imputation_m20 | 0.9908 | 0.5677 | 1.729 |
| brain | Pancreas vs Genitourinary | multiple_imputation_m20 | 2.861 | 1.75 | 4.676 |
| brain | Upper GI vs Genitourinary | multiple_imputation_m20 | 1.465 | 0.8573 | 2.504 |
| brain | Other vs Genitourinary | multiple_imputation_m20 | 0.9945 | 0.6119 | 1.616 |
| liver | Gynecologic vs Genitourinary | multiple_imputation_m20 | 0.9532 | 0.7886 | 1.152 |
| liver | Colorectal vs Genitourinary | multiple_imputation_m20 | 2.847 | 2.536 | 3.196 |
| liver | Pancreas vs Genitourinary | multiple_imputation_m20 | 3.785 | 3.38 | 4.237 |
| liver | Upper GI vs Genitourinary | multiple_imputation_m20 | 2.278 | 2 | 2.594 |
| liver | Other vs Genitourinary | multiple_imputation_m20 | 1.29 | 1.121 | 1.484 |
| lung | Gynecologic vs Genitourinary | multiple_imputation_m20 | 1.283 | 0.9714 | 1.695 |
| lung | Colorectal vs Genitourinary | multiple_imputation_m20 | 1.228 | 0.9643 | 1.564 |
| lung | Pancreas vs Genitourinary | multiple_imputation_m20 | 1.295 | 0.963 | 1.743 |
| lung | Upper GI vs Genitourinary | multiple_imputation_m20 | 1.013 | 0.7666 | 1.339 |
| lung | Other vs Genitourinary | multiple_imputation_m20 | 0.7039 | 0.5434 | 0.9119 |

PR, adjusted prevalence ratio; CI, confidence interval. complete_case uses known status for the organ analyzed; unknown_negative codes unknown status as absent; multiple_imputation_m20 pools 20 imputations. CI limits are 95% limits. Models adjust for age, sex, race/ethnicity, diagnosis year, and morphology.

**Table S5B. Numerical mixing diagnostics**

| **Organ** | **Unknown N** | **Split R-hat** | **Early mean** | **Late mean** |
| --- | --- | --- | --- | --- |
| bone | 150 | 1.0016 | 0.1027 | 0.1019 |
| brain | 155 | 1.0017 | 0.0357 | 0.0352 |
| liver | 124 | 0.9974 | 0.1445 | 0.1450 |
| lung | 174 | 1.0024 | 0.1004 | 0.1019 |

Means are imputed-positive fractions over updates 11–30 and 31–50. All 4,000 conditional logistic fits converged without warnings. R-hat summarizes imputed frequencies only; no formal claim of complete chain convergence or missing-at-random identification is made.

**Table S5C. Iteration-length sensitivity of pooled prevalence ratios**

| **Organ / site** | **10 updates PR (95% CI)** | **50 updates PR (95% CI)** | **MCSE / SE** |
| --- | --- | --- | --- |
| bone / Gynecologic | 0.627 (0.477–0.825) | 0.630 (0.479–0.830) | 0.031 |
| bone / Colorectal | 0.580 (0.463–0.727) | 0.576 (0.460–0.722) | 0.030 |
| bone / Pancreas | 0.649 (0.494–0.853) | 0.652 (0.496–0.857) | 0.039 |
| bone / Upper GI | 0.513 (0.399–0.660) | 0.512 (0.398–0.659) | 0.022 |
| bone / Other | 0.552 (0.454–0.672) | 0.555 (0.457–0.675) | 0.026 |
| brain / Gynecologic | 0.772 (0.393–1.518) | 0.760 (0.386–1.496) | 0.027 |
| brain / Colorectal | 0.963 (0.551–1.685) | 0.973 (0.556–1.704) | 0.035 |
| brain / Pancreas | 2.767 (1.697–4.514) | 2.830 (1.738–4.608) | 0.032 |
| brain / Upper GI | 1.454 (0.850–2.486) | 1.450 (0.844–2.489) | 0.041 |
| brain / Other | 0.981 (0.602–1.598) | 0.986 (0.606–1.603) | 0.031 |
| liver / Gynecologic | 0.950 (0.786–1.149) | 0.953 (0.789–1.152) | 0.021 |
| liver / Colorectal | 2.850 (2.539–3.200) | 2.852 (2.540–3.202) | 0.018 |
| liver / Pancreas | 3.783 (3.378–4.237) | 3.788 (3.383–4.242) | 0.018 |
| liver / Upper GI | 2.285 (2.006–2.602) | 2.279 (2.001–2.596) | 0.020 |
| liver / Other | 1.291 (1.121–1.486) | 1.291 (1.122–1.486) | 0.015 |
| lung / Gynecologic | 1.273 (0.963–1.682) | 1.282 (0.971–1.692) | 0.023 |
| lung / Colorectal | 1.223 (0.960–1.557) | 1.243 (0.975–1.583) | 0.031 |
| lung / Pancreas | 1.278 (0.950–1.719) | 1.298 (0.962–1.751) | 0.047 |
| lung / Upper GI | 1.009 (0.764–1.333) | 1.018 (0.770–1.345) | 0.036 |
| lung / Other | 0.702 (0.543–0.909) | 0.710 (0.548–0.920) | 0.034 |

All site contrasts use genitourinary primaries as reference. These are a separate 20-chain diagnostic rerun, not replacements for the original estimates in Table S5. MCSE/SE is shown at update 50; the maximum over both update lengths was 0.0468.

# Supplementary Table S6. Sparse interactions and proportional-hazards diagnostics

| **Covariate or interaction** | **Schoenfeld χ²** | **P value** | **P<0.05** |
| --- | --- | --- | --- |
| age10 | 49.48 | 2.006e-12 | Yes |
| male | 0.02526 | 0.8737 | No |
| met_bone | 1.025 | 0.3114 | No |
| met_brain | 2.018 | 0.1554 | No |
| met_liver | 6.146 | 0.01317 | Yes |
| met_lung | 0.00143 | 0.9698 | No |
| morph_Large-cell | 0.0005399 | 0.9815 | No |
| race_Hispanic | 0.02134 | 0.8838 | No |
| race_NHAPI | 1.763 | 0.1842 | No |
| race_NHB | 0.2643 | 0.6071 | No |
| race_Other/unknown | 1.494 | 0.2217 | No |
| site_Colorectal | 1.097 | 0.295 | No |
| site_Colorectal__x__met_bone | 0.09279 | 0.7607 | No |
| site_Colorectal__x__met_brain | 0.1936 | 0.6599 | No |
| site_Colorectal__x__met_liver | 0.2496 | 0.6174 | No |
| site_Colorectal__x__met_lung | 0.3203 | 0.5714 | No |
| site_Gynecologic | 6.003 | 0.01428 | Yes |
| site_Gynecologic__x__met_bone | 1.404 | 0.2361 | No |
| site_Gynecologic__x__met_brain | 0.1207 | 0.7283 | No |
| site_Gynecologic__x__met_liver | 4.838 | 0.02784 | Yes |
| site_Gynecologic__x__met_lung | 0.1775 | 0.6735 | No |
| site_Other | 3.907 | 0.04807 | Yes |
| site_Other__x__met_bone | 0.8543 | 0.3553 | No |
| site_Other__x__met_brain | 0.01108 | 0.9162 | No |
| site_Other__x__met_liver | 1.982 | 0.1591 | No |
| site_Other__x__met_lung | 2.048 | 0.1524 | No |
| site_Pancreas | 8.073 | 0.004494 | Yes |
| site_Pancreas__x__met_bone | 0.005754 | 0.9395 | No |
| site_Pancreas__x__met_brain | 0.4852 | 0.4861 | No |
| site_Pancreas__x__met_liver | 5.341 | 0.02083 | Yes |
| site_Pancreas__x__met_lung | 0.7269 | 0.3939 | No |
| site_Upper GI | 0.09602 | 0.7567 | No |
| site_Upper GI__x__met_bone | 0.6094 | 0.435 | No |
| site_Upper GI__x__met_brain | 0.08392 | 0.7721 | No |
| site_Upper GI__x__met_liver | 0.5052 | 0.4772 | No |
| site_Upper GI__x__met_lung | 0.3444 | 0.5573 | No |
| year5 | 1.813 | 0.1781 | No |

age10=(age−65)/10; year5=(diagnosis year−2015)/5. NHB, non-Hispanic Black; NHAPI, non-Hispanic Asian or Pacific Islander; met_, organ-involvement indicator; __x__, interaction term. P values are nominal covariate-specific tests, without multiplicity adjustment.

**Table S6B. Site-by-organ information in the interaction cohorts**

| **Site / organ** | **Full +** | **Full −** | **Early +** | **Early −** | **Later +** | **Later −** |
| --- | --- | --- | --- | --- | --- | --- |
| GU / bone | 446/393 | 266/240 | 187/183 | 118/117 | 207/188 | 115/104 |
| GU / brain | 49/46 | 663/587 | 22/22 | 283/278 | 23/21 | 299/271 |
| GU / liver | 386/348 | 326/285 | 169/167 | 136/133 | 169/155 | 153/137 |
| GU / lung | 212/193 | 500/440 | 98/98 | 207/202 | 85/79 | 237/213 |
| GYN / bone | 101/90 | 145/128 | 42/40 | 73/70 | 42/39 | 58/50 |
| GYN / brain | 17/12 | 229/206 | 6/5 | 109/105 | 7/6 | 93/83 |
| GYN / liver | 152/135 | 94/83 | 74/71 | 41/39 | 57/52 | 43/37 |
| GYN / lung | 114/102 | 132/116 | 51/49 | 64/61 | 46/41 | 54/48 |
| CR / bone | 95/85 | 404/372 | 39/39 | 159/152 | 45/43 | 200/189 |
| CR / brain | 19/16 | 480/441 | 5/4 | 193/187 | 10/9 | 235/223 |
| CR / liver | 457/427 | 42/30 | 184/178 | 14/13 | 227/217 | 18/15 |
| CR / lung | 111/99 | 388/358 | 36/34 | 162/157 | 59/57 | 186/175 |
| PAN / bone | 50/38 | 242/220 | 19/17 | 105/103 | 23/20 | 114/105 |
| PAN / brain | 25/21 | 267/237 | 9/9 | 115/111 | 11/10 | 126/115 |
| PAN / liver | 274/245 | 18/13 | 115/113 | 9/7 | 131/121 | 6/4 |
| PAN / lung | 50/44 | 242/214 | 21/20 | 103/100 | 23/21 | 114/104 |
| UGI / bone | 57/50 | 216/186 | 23/23 | 89/87 | 28/27 | 93/83 |
| UGI / brain | 19/16 | 254/220 | 8/8 | 104/102 | 6/6 | 115/104 |
| UGI / liver | 237/204 | 36/32 | 95/93 | 17/17 | 107/97 | 14/13 |
| UGI / lung | 56/45 | 217/191 | 20/19 | 92/91 | 23/21 | 98/89 |
| Other / bone | 115/104 | 219/181 | 48/47 | 84/79 | 54/49 | 111/94 |
| Other / brain | 23/17 | 311/268 | 8/7 | 124/119 | 14/10 | 151/133 |
| Other / liver | 250/220 | 84/65 | 95/91 | 37/35 | 129/120 | 36/23 |
| Other / lung | 73/61 | 261/224 | 28/27 | 104/99 | 35/27 | 130/116 |

Cells show patients/deaths, not independent effective sample sizes; patients can contribute to several organ rows. + and − indicate recorded organ status. Full includes all eligible years; early=2010–2017; later=2018–2022. GU, genitourinary; GYN, gynecologic; CR, colorectal; PAN, pancreatic; UGI, upper gastrointestinal. The 37-coefficient interaction model used 2,356 patients/2,087 deaths overall (56.4 deaths per coefficient) and 986/957 in the early period (25.9 per coefficient). These aggregate ratios do not measure information for individual interactions. The separate parsimonious prediction-development cohort had 1,297 patients/1,241 deaths.

**Table S6C. Interaction coefficient penalty profile — Full**

| **Interaction** | **0** | **0.001** | **0.01** | **0.1** | **1** |
| --- | --- | --- | --- | --- | --- |
| GYN × bone | 0.0227 | 0.0235 | 0.0322 | 0.0710 | 0.0357 |
| GYN × brain | -0.4620 | -0.4596 | -0.4381 | -0.2933 | -0.0613 |
| GYN × liver | -0.2091 | -0.2073 | -0.1884 | -0.0761 | 0.0092 |
| GYN × lung | 0.0201 | 0.0216 | 0.0352 | 0.0892 | 0.0462 |
| CR × bone | 0.2454 | 0.2446 | 0.2406 | 0.2236 | 0.1219 |
| CR × brain | -0.3915 | -0.3914 | -0.3870 | -0.3122 | -0.1233 |
| CR × liver | 0.3507 | 0.3448 | 0.3064 | 0.1991 | 0.0910 |
| CR × lung | -0.0759 | -0.0760 | -0.0743 | -0.0434 | 0.0217 |
| PAN × bone | -0.3958 | -0.3962 | -0.3942 | -0.3278 | -0.1146 |
| PAN × brain | 0.2513 | 0.2500 | 0.2441 | 0.2413 | 0.1308 |
| PAN × liver | 0.6027 | 0.5807 | 0.4531 | 0.2083 | 0.0652 |
| PAN × lung | 0.0917 | 0.0877 | 0.0661 | 0.0508 | 0.0687 |
| UGI × bone | 0.1475 | 0.1496 | 0.1664 | 0.1947 | 0.0819 |
| UGI × brain | 0.0111 | 0.0159 | 0.0535 | 0.1844 | 0.1408 |
| UGI × liver | -0.4730 | -0.4644 | -0.3958 | -0.1540 | -0.0158 |
| UGI × lung | 0.0070 | 0.0092 | 0.0267 | 0.0838 | 0.0726 |
| Other × bone | 0.4229 | 0.4208 | 0.4057 | 0.3162 | 0.0934 |
| Other × brain | -0.4463 | -0.4467 | -0.4470 | -0.4071 | -0.2288 |
| Other × liver | 0.2746 | 0.2703 | 0.2398 | 0.1238 | 0.0302 |
| Other × lung | 0.2183 | 0.2162 | 0.2003 | 0.1213 | 0.0086 |

Values are log-hazard interaction coefficients, not stratum-specific HRs. Columns are lifelines penalizer values, with all 37 coefficients penalized. The grid is a sensitivity assessment, not a tuned replacement for the original analysis; no inferential P values are assigned to penalized coefficients.

**Table S6C. Interaction coefficient penalty profile — 2010-2017**

| **Interaction** | **0** | **0.001** | **0.01** | **0.1** | **1** |
| --- | --- | --- | --- | --- | --- |
| GYN × bone | 0.0026 | 0.0065 | 0.0345 | 0.1014 | 0.0448 |
| GYN × brain | -0.0904 | -0.0876 | -0.0653 | 0.0342 | 0.0775 |
| GYN × liver | -0.3382 | -0.3316 | -0.2821 | -0.0953 | 0.0136 |
| GYN × lung | -0.0015 | 0.0019 | 0.0264 | 0.0978 | 0.0579 |
| CR × bone | 0.2917 | 0.2946 | 0.3123 | 0.3191 | 0.1693 |
| CR × brain | -1.0059 | -0.9963 | -0.9338 | -0.7558 | -0.4062 |
| CR × liver | -0.0542 | -0.0449 | 0.0116 | 0.0941 | 0.0630 |
| CR × lung | -0.1955 | -0.1932 | -0.1775 | -0.1171 | -0.0028 |
| PAN × bone | -0.4864 | -0.4815 | -0.4466 | -0.3099 | -0.0685 |
| PAN × brain | 0.4721 | 0.4736 | 0.4820 | 0.4681 | 0.2412 |
| PAN × liver | 0.8093 | 0.7893 | 0.6586 | 0.3266 | 0.1048 |
| PAN × lung | 0.2664 | 0.2610 | 0.2242 | 0.1155 | 0.0515 |
| UGI × bone | 0.0804 | 0.0866 | 0.1295 | 0.2079 | 0.0919 |
| UGI × brain | 0.1190 | 0.1272 | 0.1862 | 0.3494 | 0.2473 |
| UGI × liver | -0.6136 | -0.6004 | -0.5037 | -0.1975 | -0.0223 |
| UGI × lung | 0.0055 | 0.0080 | 0.0253 | 0.0721 | 0.0842 |
| Other × bone | 0.5044 | 0.5054 | 0.5083 | 0.4347 | 0.1393 |
| Other × brain | -0.5278 | -0.5250 | -0.5045 | -0.4142 | -0.2242 |
| Other × liver | -0.0535 | -0.0511 | -0.0360 | -0.0180 | -0.0151 |
| Other × lung | 0.3774 | 0.3779 | 0.3794 | 0.3355 | 0.1359 |

Values are log-hazard interaction coefficients, not stratum-specific HRs. Columns are lifelines penalizer values, with all 37 coefficients penalized. The grid is a sensitivity assessment, not a tuned replacement for the original analysis; no inferential P values are assigned to penalized coefficients.

**Table S6D. Rank-Schoenfeld tests after model specification**

| **Model** | **Term** | **Chi-square** | **df** | **P** |
| --- | --- | --- | --- | --- |
| Full interaction | age10 | 43.602 | 1 | 4.024e-11 |
| Full interaction | year5 | 2.450 | 1 | 0.1175 |
| Full interaction | male | 0.999 | 1 | 0.3176 |
| Full interaction | race | 5.004 | 4 | 0.2869 |
| Full interaction | large | 5.884 | 1 | 0.01528 |
| Full interaction | site | 20.605 | 5 | 0.0009615 |
| Full interaction | bone | 23.266 | 1 | 1.411e-06 |
| Full interaction | brain | 6.075 | 1 | 0.01371 |
| Full interaction | liver | 12.066 | 1 | 0.0005136 |
| Full interaction | lung | 0.501 | 1 | 0.4792 |
| Full interaction | site:bone | 6.872 | 5 | 0.2303 |
| Full interaction | site:brain | 1.307 | 5 | 0.9342 |
| Full interaction | site:liver | 16.200 | 5 | 0.006295 |
| Full interaction | site:lung | 12.661 | 5 | 0.02677 |
| Full interaction | GLOBAL | 122.296 | 37 | 4.704e-11 |
| Piecewise | age10 | 6.509 | 1 | 0.01073 |
| Piecewise | year5 | 1.814 | 1 | 0.178 |
| Piecewise | male | 0.116 | 1 | 0.7333 |
| Piecewise | race | 3.984 | 4 | 0.4082 |
| Piecewise | large | 0.597 | 1 | 0.4397 |
| Piecewise | site | 5.169 | 5 | 0.3956 |
| Piecewise | bone | 7.241 | 1 | 0.007127 |
| Piecewise | brain | 3.447 | 1 | 0.06338 |
| Piecewise | liver | 10.502 | 1 | 0.001193 |
| Piecewise | lung | 0.989 | 1 | 0.3199 |
| Piecewise | Age × late | 0.007 | 1 | 0.9322 |
| Piecewise | GYN × late | 0.691 | 1 | 0.4059 |
| Piecewise | PAN × late | 0.164 | 1 | 0.6852 |
| Piecewise | Other × late | 0.000 | 1 | 0.9911 |
| Piecewise | Liver × late | 3.277 | 1 | 0.07026 |
| Piecewise | Liver × late × GYN | 0.009 | 1 | 0.9246 |
| Piecewise | Liver × late × CR | 1.789 | 1 | 0.1811 |
| Piecewise | Liver × late × PAN | 0.804 | 1 | 0.37 |
| Piecewise | Liver × late × UGI | 0.617 | 1 | 0.4322 |
| Piecewise | Liver × late × Other | 0.498 | 1 | 0.4806 |
| Piecewise | site:liver | 5.680 | 5 | 0.3386 |
| Piecewise | GLOBAL | 64.320 | 32 | 0.0006039 |
| M0 analogue | Large-cell | 1.656 | 1 | 0.1982 |
| M0 analogue | Age spline 1 | 9.335 | 1 | 0.002248 |
| M0 analogue | Age spline 2 | 1.478 | 1 | 0.224 |
| M0 analogue | Age spline 3 | 0.924 | 1 | 0.3364 |
| M0 analogue | Male | 0.245 | 1 | 0.6208 |
| M0 analogue | GLOBAL | 22.586 | 5 | 0.000405 |
| M1 analogue | Large-cell | 2.294 | 1 | 0.1298 |
| M1 analogue | GYN | 0.114 | 1 | 0.7355 |
| M1 analogue | CR | 1.475 | 1 | 0.2245 |
| M1 analogue | PAN | 3.504 | 1 | 0.06121 |
| M1 analogue | UGI | 0.278 | 1 | 0.5978 |
| M1 analogue | Other | 1.404 | 1 | 0.2361 |
| M1 analogue | Age spline 1 | 10.484 | 1 | 0.001204 |
| M1 analogue | Age spline 2 | 1.563 | 1 | 0.2112 |
| M1 analogue | Age spline 3 | 1.072 | 1 | 0.3004 |
| M1 analogue | Male | 0.272 | 1 | 0.6017 |
| M1 analogue | GLOBAL | 45.463 | 10 | 1.794e-06 |
| M2 analogue | Large-cell | 3.116 | 1 | 0.07753 |
| M2 analogue | GYN | 0.000 | 1 | 0.9883 |
| M2 analogue | CR | 2.310 | 1 | 0.1285 |
| M2 analogue | PAN | 3.859 | 1 | 0.04948 |
| M2 analogue | UGI | 0.168 | 1 | 0.682 |
| M2 analogue | Other | 1.258 | 1 | 0.2621 |
| M2 analogue | Organ count 1 | 5.307 | 1 | 0.02125 |
| M2 analogue | Organ count 2 | 0.174 | 1 | 0.6765 |
| M2 analogue | Organ count 3+ | 9.816 | 1 | 0.00173 |
| M2 analogue | Age spline 1 | 11.033 | 1 | 0.0008949 |
| M2 analogue | Age spline 2 | 1.091 | 1 | 0.2963 |
| M2 analogue | Age spline 3 | 0.648 | 1 | 0.421 |
| M2 analogue | Male | 0.211 | 1 | 0.6459 |
| M2 analogue | GLOBAL | 55.578 | 13 | 3.198e-07 |
| M3 analogue | Large-cell | 3.153 | 1 | 0.07581 |
| M3 analogue | GYN | 0.006 | 1 | 0.9382 |
| M3 analogue | CR | 2.511 | 1 | 0.1131 |
| M3 analogue | PAN | 3.439 | 1 | 0.06367 |
| M3 analogue | UGI | 0.092 | 1 | 0.7619 |
| M3 analogue | Other | 1.472 | 1 | 0.225 |
| M3 analogue | Age spline 1 | 10.415 | 1 | 0.00125 |
| M3 analogue | Age spline 2 | 1.472 | 1 | 0.2251 |
| M3 analogue | Age spline 3 | 0.612 | 1 | 0.4341 |
| M3 analogue | Male | 0.454 | 1 | 0.5006 |
| M3 analogue | bone | 15.851 | 1 | 6.853e-05 |
| M3 analogue | brain | 0.368 | 1 | 0.544 |
| M3 analogue | liver | 4.624 | 1 | 0.03153 |
| M3 analogue | lung | 0.145 | 1 | 0.7032 |
| M3 analogue | GLOBAL | 54.282 | 14 | 1.15e-06 |

R cox.zph tests use the rank transform. Full interaction and piecewise fits use Efron ties; prediction analogues use Breslow ties and one redundant spline column omitted. Spline coordinates refer to the basis in Table S11C. Tests are nominal and exploratory. Prediction analogues are unpenalized; these are not formal tests of ridge estimators. age10 and year5 are scaled age and year; late denotes the interval after six months. Grouped factor tests may have more than one degree of freedom.

# Supplementary Table S7. Exploratory time-varying liver associations

| Primary site | Interval | Adjusted HR (95% CI) |
| --- | --- | --- |
| Genitourinary | 0-6 months | 1.99 (1.59–2.50) |
| Genitourinary | After 6 months | 1.59 (1.27–1.98) |
| Gynecologic | 0-6 months | 1.07 (0.73–1.58) |
| Gynecologic | After 6 months | 1.73 (1.17–2.56) |
| Colorectal | 0-6 months | 3.45 (2.29–5.20) |
| Colorectal | After 6 months | 2.05 (1.37–3.06) |
| Pancreas | 0-6 months | 1.79 (0.87–3.68) |
| Pancreas | After 6 months | 3.71 (1.50–9.20) |
| Upper GI | 0-6 months | 1.27 (0.84–1.90) |
| Upper GI | After 6 months | 0.69 (0.45–1.06) |
| Other | 0-6 months | 1.78 (1.20–2.64) |
| Other | After 6 months | 2.13 (1.42–3.18) |

Likelihood-ratio test for six liver time terms: χ²=21.9647, df=6, P=0.001229. Estimates are conditional on survival to the corresponding interval and should not be interpreted causally. HR, hazard ratio; CI, confidence interval.

# Supplementary Table S8. Liver associations with 12-month mortality

| Primary site | Adjusted OR (95% CI) |
| --- | --- |
| Genitourinary | 3.44 (2.30–5.13) |
| Gynecologic | 2.35 (1.15–4.82) |
| Colorectal | 5.54 (2.34–13.11) |
| Pancreas | 7.72 (1.65–36.08) |
| Upper GI | 1.16 (0.40–3.42) |
| Other | 4.45 (2.17–9.14) |

N=2,203; deaths by 12 months=1,676. Full site-by-organ logistic model. Earlier-censored survivors were excluded; these are conditional odds ratios, not risk ratios or hazard ratios. The global 20-df interaction test gave P=0.00793. OR, odds ratio; CI, confidence interval.

# Supplementary Table S9. Exclusion of stage-discordant organ-positive cases

| Analysis | N / deaths | Interaction χ² (20 df) | P |
| --- | --- | --- | --- |
| Original core cohort | 2,356 / 2,087 | 43.4857 | 0.001763 |
| Exclude eight discordant records | 2,348 / 2,080 | 45.7321 | 0.000877 |

Discordant records had localized stage (n=1), regional direct extension (n=2), or regional direct extension plus nodes (n=5), despite a positive recorded organ. Their exclusion did not remove the global interaction evidence.

# Supplementary Table S10. Paired bootstrap comparison of M3 and M2

| **metric** | **estimate** | **ci_low** | **ci_high** | **bootstrap_successes** |
| --- | --- | --- | --- | --- |
| M3 - M2 Harrell C-index | 0.008509 | 0.003524 | 0.01389 | 500 |
| M3 - M2 12-month AUC | 0.011885 | 0.003301 | 0.02306 | 500 |
| M3 - M2 12-month Brier score | -0.002015 | -0.003767 | -0.0003925 | 500 |

Estimates are original-sample M3 minus M2 differences. CI limits are 95% paired percentile bootstrap limits from 500 patient-level resamples with fixed fitted models. A positive C-index/AUC difference or negative Brier-score difference favors M3.

# Supplementary Table S11. Penalization, coefficient shrinkage, and sparse-model sensitivity

| **Analysis** | **Finding** |
| --- | --- |
| M0 Basic | Selected ridge penalty strength λ=0.000001; 6 design columns (rank 5) |
| M1 Site | Selected ridge penalty strength λ=15.8489; 11 design columns (rank 10) |
| M2 Four-organ count | Selected ridge penalty strength λ=0.000001; 14 design columns (rank 13) |
| M3 Spectrum | Selected ridge penalty strength λ=39.8107; 15 design columns (rank 14); implemented as software parameter alpha |
| Elastic-net sensitivity | Mixing parameter 0.5; selected alpha 0.0138699 by five-fold mean 6/12/24-month Brier score; 14 of 15 coefficients nonzero. |
| Performance | Elastic net: C-index 0.6062; 12-month AUC 0.6718; 12-month Brier score 0.1964; calibration slope 0.9498. |
| Table 4 confidence intervals | Performance intervals from 300 later-period patient bootstrap samples; coefficients and penalty fixed. These do not include model-selection uncertainty. |
| M3 versus M2 paired bootstrap | Identical later-period patient indices were applied to fixed M2 and M3 predictions |
| Interpretation | Penalty magnitude alone does not measure proportional coefficient shrinkage. Predictor design columns are standardized before fitting; model-level and coefficient-level comparisons are reported in Tables S11B–C. |

**Table S11B. Model-level shrinkage**

| **Model** | **Alpha** | **Columns / rank** | **Norm reduction %** | **LP SD reduction %** |
| --- | --- | --- | --- | --- |
| M0 Basic | 1e-06 | 6 / 5 | 0.0000 | 0.0000 |
| M1 Site | 15.8489 | 11 / 10 | 2.1969 | 1.2831 |
| M2 Count | 1e-06 | 14 / 13 | 0.0000 | 0.0000 |
| M3 Spectrum | 39.8107 | 15 / 14 | 4.9366 | 3.2997 |

All models use 1,297 development patients and 1,241 deaths. Norms use standardized coefficients in the same minimum-norm spline basis. LP is the development linear predictor. Values rounding to 0.0000% indicate negligible, not mathematically zero, shrinkage. Rank counts independent design contrasts, not effective ridge degrees of freedom.

**Table S11C. Coefficient comparison on the original predictor scale**

| **Model** | **Predictor** | **Unpenalized β** | **Ridge β** | **Magnitude reduction %** |
| --- | --- | --- | --- | --- |
| M0 | Large-cell | 0.1082 | 0.1082 | 0.00 |
| M0 | Age spline 1 | -0.1793 | -0.1793 | 0.00 |
| M0 | Age spline 2 | -0.0874 | -0.0874 | 0.00 |
| M0 | Age spline 3 | -0.0131 | -0.0131 | 0.00 |
| M0 | Age spline 4 | 0.4201 | 0.4201 | -0.00 |
| M0 | Male | 0.0639 | 0.0639 | 0.00 |
| M1 | Large-cell | -0.0192 | -0.0153 | 20.70 |
| M1 | Gynecologic | 0.0471 | 0.0371 | 21.24 |
| M1 | Colorectal | 0.3553 | 0.3442 | 3.13 |
| M1 | Pancreas | 0.3100 | 0.2993 | 3.47 |
| M1 | Upper GI | 0.1121 | 0.1060 | 5.44 |
| M1 | Other | -0.1841 | -0.1878 | -2.00 |
| M1 | Age spline 1 | -0.1968 | -0.1930 | 1.91 |
| M1 | Age spline 2 | -0.0975 | -0.0964 | 1.19 |
| M1 | Age spline 3 | 0.0073 | 0.0067 | — |
| M1 | Age spline 4 | 0.4222 | 0.4167 | 1.31 |
| M1 | Male | 0.0962 | 0.0916 | 4.82 |
| M2 | Large-cell | -0.0318 | -0.0318 | 0.00 |
| M2 | Gynecologic | 0.2272 | 0.2272 | 0.00 |
| M2 | Colorectal | 0.3863 | 0.3863 | 0.00 |
| M2 | Pancreas | 0.3779 | 0.3779 | 0.00 |
| M2 | Upper GI | 0.2227 | 0.2227 | 0.00 |
| M2 | Other | -0.0116 | -0.0116 | -0.00 |
| M2 | 1 | 0.3808 | 0.3808 | 0.00 |
| M2 | 2 | 0.7371 | 0.7371 | 0.00 |
| M2 | 3+ | 1.0052 | 1.0052 | 0.00 |
| M2 | Age spline 1 | -0.2228 | -0.2228 | -0.00 |
| M2 | Age spline 2 | -0.1114 | -0.1114 | -0.00 |
| M2 | Age spline 3 | 0.0062 | 0.0062 | — |
| M2 | Age spline 4 | 0.4844 | 0.4844 | 0.00 |
| M2 | Male | 0.1264 | 0.1264 | 0.00 |
| M3 | Large-cell | -0.0433 | -0.0331 | 23.65 |
| M3 | Gynecologic | 0.1837 | 0.1458 | 20.63 |
| M3 | Colorectal | 0.2670 | 0.2402 | 10.05 |
| M3 | Pancreas | 0.2544 | 0.2268 | 10.84 |
| M3 | Upper GI | 0.1144 | 0.0959 | 16.22 |
| M3 | Other | -0.0793 | -0.0984 | -24.19 |
| M3 | Age spline 1 | -0.2283 | -0.2162 | 5.31 |
| M3 | Age spline 2 | -0.0991 | -0.0958 | 3.32 |
| M3 | Age spline 3 | 0.0007 | -0.0007 | — |
| M3 | Age spline 4 | 0.4719 | 0.4542 | 3.74 |
| M3 | Male | 0.1324 | 0.1167 | 11.87 |
| M3 | bone | 0.2046 | 0.1918 | 6.24 |
| M3 | brain | 0.2526 | 0.2439 | 3.47 |
| M3 | liver | 0.5270 | 0.5098 | 3.26 |
| M3 | lung | 0.2858 | 0.2771 | 3.06 |

β is a log-hazard coefficient. References: female, small-cell morphology, genitourinary primary, organ absent, or count=0. Spline coordinates are basis-dependent and not separately interpretable age effects. Comparisons use the minimum-norm unpenalized representation described above. A negative reduction denotes increased magnitude; ratios are omitted when |unpenalized β|≤0.01. M3 categorical coefficients had no sign changes. This is a coefficient comparison, not a confidence-interval table.

# Supplementary Table S12. Later-period performance before and during the pandemic era

| **period** | **n** | **events** | **harrell_c** | **auc_12m** | **brier_12m** | **observed_12m_mortality** | **mean_predicted_12m_mortality** |
| --- | --- | --- | --- | --- | --- | --- | --- |
| 2018-2019 | 523 | 494 | 0.6158 | 0.6747 | 0.186 | 0.725382 | 0.7089 |
| 2020-2022 | 851 | 720 | 0.6031 | 0.6736 | 0.2025 | 0.677234 | 0.7151 |

Events are deaths over all observed follow-up, not only within 12 months. Observed 12-month mortality is one minus Kaplan–Meier survival. AUC, cumulative/dynamic area under the curve; Brier scores use inverse probability of censoring weights.

# Supplementary Table S13. Recorded first-course treatment by period

| **period** | **variable** | **category** | **n** | **denominator** | **pct** |
| --- | --- | --- | --- | --- | --- |
| 2010-2017 | Cohort size | All | 1297 | 1297 | 100 |
| 2010-2017 | Chemotherapy | Yes | 884 | 1297 | 68.16 |
| 2010-2017 | Radiotherapy | Yes | 319 | 1297 | 24.6 |
| 2010-2017 | Primary-site surgery | Yes | 457 | 1296 | 35.26 |
| 2018-2019 | Cohort size | All | 523 | 523 | 100 |
| 2018-2019 | Chemotherapy | Yes | 358 | 523 | 68.45 |
| 2018-2019 | Radiotherapy | Yes | 123 | 523 | 23.52 |
| 2018-2019 | Primary-site surgery | Yes | 153 | 522 | 29.31 |
| 2020-2022 | Cohort size | All | 851 | 851 | 100 |
| 2020-2022 | Chemotherapy | Yes | 587 | 851 | 68.98 |
| 2020-2022 | Radiotherapy | Yes | 171 | 851 | 20.09 |
| 2020-2022 | Primary-site surgery | Yes | 257 | 851 | 30.2 |

Percentages use the displayed denominator. Surgery denominators exclude one unknown record in each of 2010–2017 and 2018–2019. Chemotherapy and radiotherapy indicate recorded treatment; absent or unknown records do not establish that treatment was not received.

# Supplementary Figure 1. Construction of the strict EP-PD-NEC cohort and analysis populations

The SEER export was restricted to strict morphology codes, known nonpulmonary primary sites, microscopically confirmed tumors, non-death-certificate/autopsy cases, consistent invasive-stage records, first malignant primaries, and valid survival information. The branches account for complete versus incomplete four-organ status, the core metastatic-spectrum cohort, and the distant-stage prognostic cohort and calendar-period subsets. Of 3,018 prediction-eligible cases, 347 diagnosed in 2023 were excluded, leaving 2,671 across the two modeling periods. Eight organ-positive cases had localized/regional stage and were included only in the core cohort; exclusion sensitivity is in Table S9. The two analysis populations overlap and are not disjoint branches.

# Supplementary Figure 2. Full primary-site-by-organ prognostic interaction matrix

Adjusted HRs and 95% CIs for bone, brain, liver, and lung metastases within each primary-site group from the 20-df Cox interaction model. Filled markers indicate BH-FDR<0.05 across the 24 exploratory stratum-specific effects. The pooled global interaction likelihood-ratio test gives χ²=43.49 with 20 df (P=0.00176). Models adjusted for age, sex, race/ethnicity, diagnosis year, morphology, primary site, all four organ indicators, and all site-by-organ interactions. HR, hazard ratio; CI, confidence interval; BH-FDR, Benjamini–Hochberg false discovery rate.

# Supplementary Figure 3. Interpretation of the selected metastatic-spectrum model

(A) Partial age effect from the four-knot restricted cubic spline, expressed as relative hazard versus age 65 while holding other predictors constant. (B) Exponentiated shrunken M3 coefficients for categorical predictors. Only point estimates of the penalized coefficients are displayed. Reference categories are female sex, small-cell morphology, genitourinary primary site, and absence of the corresponding organ metastasis.
